# Five major shifts of diversification through the long evolutionary history of Magnoliidae (angiosperms)

**DOI:** 10.1186/s12862-015-0320-6

**Published:** 2015-03-18

**Authors:** Julien Massoni, Thomas LP Couvreur, Hervé Sauquet

**Affiliations:** Laboratoire Ecologie, Systématique, Evolution, Université Paris-Sud, CNRS UMR 8079, 91405 Orsay, France; Institut de Recherche pour le Développement (IRD), UMR-DIADE, 911, avenue Agropolis, BP 64501, Cedex 5, F-34394 Montpellier, France; Département des Sciences Biologiques, Université de Yaoundé I, Ecole Normale Supérieure, Laboratoire de Botanique systématique et d’Ecologie, B.P. 047 Yaoundé, Cameroon

**Keywords:** Angiosperms, Magnoliidae, Molecular dating, Diversification, Speciation, Extinction

## Abstract

**Background:**

With 10,000 species, Magnoliidae are the largest clade of flowering plants outside monocots and eudicots. Despite an ancient and rich fossil history, the tempo and mode of diversification of Magnoliidae remain poorly known. Using a molecular data set of 12 markers and 220 species (representing >75% of genera in Magnoliidae) and six robust, internal fossil age constraints, we estimate divergence times and significant shifts of diversification across the clade. In addition, we test the sensitivity of magnoliid divergence times to the choice of relaxed clock model and various maximum age constraints for the angiosperms.

**Results:**

Compared with previous work, our study tends to push back in time the age of the crown node of Magnoliidae (178.78-126.82 million years, Myr), and of the four orders, Canellales (143.18-125.90 Myr), Piperales (158.11-88.15 Myr), Laurales (165.62-112.05 Myr), and Magnoliales (164.09-114.75 Myr). Although families vary in crown ages, Magnoliidae appear to have diversified into most extant families by the end of the Cretaceous. The strongly imbalanced distribution of extant diversity within Magnoliidae appears to be best explained by models of diversification with 6 to 13 shifts in net diversification rates. Significant increases are inferred within Piperaceae and Annonaceae, while the low species richness of Calycanthaceae, Degeneriaceae, and Himantandraceae appears to be the result of decreases in both speciation and extinction rates.

**Conclusions:**

This study provides a new time scale for the evolutionary history of an important, but underexplored, part of the tree of angiosperms. The ages of the main clades of Magnoliidae (above the family level) are older than previously thought, and in several lineages, there were significant increases and decreases in net diversification rates. This study is a new robust framework for future investigations of trait evolution and of factors influencing diversification in this group as well as angiosperms as a whole.

**Electronic supplementary material:**

The online version of this article (doi:10.1186/s12862-015-0320-6) contains supplementary material, which is available to authorized users.

## Background

Understanding the diversification history of species-rich clades is a major goal in evolutionary biology [1] as they provide important insights into the evolution of life on earth. With over 10,000 plant species, Magnoliidae, *sensu* Cantino et al. [2], are the largest clade of flowering plants outside monocots and eudicots and comprise four orders: Canellales (123 spp.), Laurales (3874 spp., incl. avocado), Magnoliales (2978 spp., incl. magnolias), and Piperales (3190 spp., incl. black pepper). Investigating the diversification of Magnoliidae is of primary importance to provide guidance for future hypotheses on the drivers of diversification of flowering plants as a whole. In addition, many organisms (incl. various butterfly and beetle groups) are highly dependent on this group for feeding or reproduction [3,4], and magnoliid species are an important part of tropical ecosystems [5,6]. As a consequence, investigating their tempo and mode of diversification will provide key knowledge to understand not only the evolutionary history of Magnoliidae as a whole, but also that of these related groups [7] and of the environments they live in [8].

It has often been suggested that the biology of angiosperms has a great influence on their own diversification [9,10]. The biology of Magnoliidae differs from that of eudicots and monocots in several respects. For instance, most members of Magnoliidae are mainly pollinated by beetles, flies, or thrips, while, in contrast to eudicots and monocots, bee or wind pollination are rare [3,11]. In addition, floral and vegetative morphology of Magnoliidae is highly variable and several of these variable traits have been shown to be associated with variation of diversification rates in other flowering plant lineages [10].

Previous molecular dating studies at large (e.g., angiosperms) or narrow (e.g., a particular family) taxonomic scale have suggested that the crown node age of Magnoliidae is older than the crown nodes of both eudicots and monocots, ranging from 240.16 to 85.07 Myr [12,13], and that several of the 19 families of Magnoliidae appear to have originated before the end of the Cretaceous [13-22]. In addition, the ages of numerous nodes within Magnoliidae have yet to be estimated [23]. In parallel, few studies have focused on the diversification of the group, being either at the scale of all angiosperms [24] or within families [25,26].

In the present study, we provide for the first time a complete time scale for Magnoliidae at the familial level. We then use this new time scale to test for significant diversification rate shifts during the evolutionary history of the clade. To do so, we take advantage of a recent improvement in the phylogeny of the group [16,27-35] and a new revision of the rich fossil record of Magnoliidae providing reliable minimum-age calibration points [36]. In order to conduct these analyses for nodes above and below the family level, we used a dataset including more than 75% of the existing genera and 12 molecular markers from the three plant genomes (6 plastid, 4 mitochondrial, 2 nuclear).

## Results

### Temporal analyses

All runs in each of the five BEAST analyses with different calibration scheme (angio-130, −140, −150, −170 and −200) converged effectively. The concatenated post-burn-in samples of the four runs in each analyses showed final effective sampling sizes (ESS) of the likelihood far above 200 [see Additional file 1]. Except for the position of Siparunaceae (Laurales) and Magnoliaceae (Magnoliales), which remained unresolved in all analyses, the great majority of the relationships among the families received high support values [see Additional file 2] and is similar in all analyses [see Additional file 3].

Conducting BEAST analyses without molecular data highlighted the non-uniformity of the temporal constraints effectively used in the analyses (results not shown). However, no effective temporal priors violated the paleontological information used to calibrate the present analyses. In addition, the temporal posterior distributions of calibrated nodes obtained with the molecular data differ from the effective temporal prior distributions obtained without these data. These mismatches for calibrated nodes suggest that molecular data are well informative.

**Figure 1:**
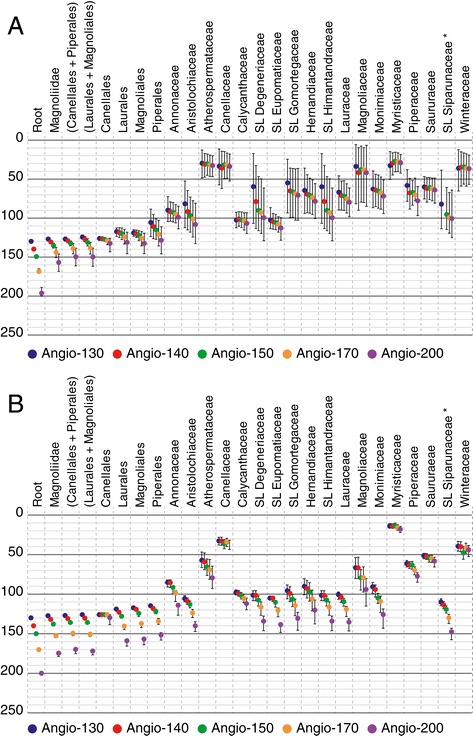
**Age estimates for the root and different nodes within the tree of Magnoliidae.** Estimates are in million years, and the root is constrained with a maximum age. Names of families refer to the crown node ages except when SL (stem lineage) is mentioned. **A**, BEAST analyses with mean age estimates and 95% credibility intervals. **B**, r8s (penalized likelihood) analyses with best-scoring ML tree age and 95% credibility intervals. *The stem lineage of Siparunaceae, in the BEAST analysis with the maximum age of angiosperms set to 140 million years, did not correspond to the same node in other analyses. Abbreviation: SL, stem lineage.

The 95% credibility intervals of the ages obtained with BEAST are larger than those obtained with r8s (Figure 1). When the maximum age of the root varied, only the ages estimated for the crown node of Magnoliidae, and the splits between Canellales and Piperales, and between Laurales and Magnoliales, are significantly different in the BEAST analyses (Figure 1A). On the contrary, in the PL analyses, the majority of age estimates are significantly different when the maximum age of the root is changed (Figure 1B).

In general, the ages estimated in BEAST and r8s were similar (Figure 1, Table 1). In the present paper, we take into account the overlapping 95% credibility intervals of all 10 analyses (see Discussion). Our results support an origin of extant Magnoliidae (crown node) between 178.78 and 126.82 Myr, that is, in the Lower Cretaceous or the Jurassic. The crown nodes of Canellales, Laurales, Magnoliales, and Piperales are dated to 143.18-125.90 Myr, 165.62-112.05 Myr, 164.09-114.75 Myr, and 158.11-88.15 Myr, respectively. By the end of the Cretaceous (66 Myr), most families (crown nodes) were probably present, with the exception of Canellaceae, Myristicaceae and Winteraceae. The oldest families in terms of crown node age are Aristolochiaceae and Calycanthaceae (BEAST: 132.66-52.38 Myr, 119.79-91.65 Myr, respectively) or Aristolochiaceae and Lauraceae (PL: 147.26-101.83 Myr, 146.61-97.3 Myr, respectively), depending on the relaxed clock method used (Figure 1, Table 1).

**Table 1:** **Age estimates for selected nodes of Magnoliidae**

	**Angio 130**	**Angio 140**	**Angio 150**	**Angio 170**	**Angio 200**
**Node**	95% CI (mean or PL)	95% IC (mean or PL)	95% CI (mean or PL)	95% CI (mean or PL)	95% CI (mean or PL)
Root	**130.10-129.81 (130.00)**	**140.10-138.90 (139.69)**	**150.10-147.70 (149.28)**	**170.10-164.95 (168.33)**	**200.10-188.92 (196.22)**
	130.00-130.00 (130.00)	140.00-140.00 (140.00)	150.00-150.00 (150.00)	170.00-170.00 (170.00)	200.00-200.00 (200.00)
Magnoliidae	**127.52-126.82 (127.15)**	**132.41-129.03 (130.72)**	**138.36-132.07 (135.33)**	**150.72-137.47 (144.12)**	**168.29-146.07 (156.83)**
	127.43-127.03 (127.22)	132.81-131.29 (131.91)	139.34-136.86 (137.74)	155.25-151.11 (152.76)	178.78-172.60 (174.56)
(Canellales + Piperales)	**127.09-126.54 (126.80)**	**130.66-127.72 (129.13)**	**135.58-129.24 (132.43)**	**145.83-132.36 (139.08)**	**161.18-138.44 (149.75)**
	127.18-126.77 (126.96)	131.77-130.09 (130.86)	137.62-134.72 (135.87)	152.54-147.45 (149.46)	175.20-167.36 (169.77)
(Laurales + Magnoliales)	**126.88-121.34 (124.27)**	**130.33-123.60 (127.24)**	**135.68-126.46 (131.13)**	**145.99-131.05 (138.43)**	**161.65-138.41 (149.70)**
	126.49-125.38 (125.88)	131.35-129.41 (130.30)	137.76-134.95 (135.95)	153.51-149.08 (150.58)	176.78-170.37 (171.89)
Canellales	**126.19-125.90 (126.00)**	**127.30-125.90 (126.38)**	**129.27-125.90 (127.07)**	**134.13-125.90 (128.84)**	**143.18-125.90 (132.42)**
	125.90-125.90 (125.90)	125.90-125.90 (125.90)	125.90-125.90 (125.90)	125.90-125.90 (125.90)	138.60-125.90 (129.79)
Laurales	**123.02-112.05 (117.38)**	**124.75-112.17 (118.46)**	**127.58-112.53 (119.91)**	**133.16-114.48 (123.95)**	**145.59-117.94 (131.11)**
	120.62-117.82 (118.99)	124.89-121.37 (122.77)	130.52-126.40 (127.87)	144.69-138.49 (140.28)	165.62-156.80 (158.93)
Magnoliales	**123.14-114.75 (118.85)**	**124.76-115.31 (120.04)**	**128.72-116.08 (122.06)**	**135.59-117.27 (126.00)**	**145.66-119.31 (132.36)**
	118.86-116.02 (117.34)	121.83-117.67 (119.22)	128.29-122.61 (124.37)	142.34-134.77 (137.02)	164.09-154.80 (156.82)
Piperales	**123.79-88.15 (105.60)**	**126.33-95.33 (110.81)**	**127.10-100.97 (114.94)**	**135.65-102.71 (120.74)**	**145.79-111.08 (128.58)**
	117.14-112.33 (114.66)	120.77-115.46 (118.03)	124.86-118.94 (122.08)	138.16-130.66 (134.08)	158.11-148.47 (151.64)
Annonaceae	**104.30-72.42 (89.75)**	**104.02-79.10 (91.71)**	**104.89-80.10 (92.64)**	**106.11-82.58 (94.96)**	**113.70-84.78 (98.94)**
	90.88-81.99 (85.34)	91.69-81.83 (85.37)	99.65-89.54 (91.38)	109.21-95.39 (98.62)	126.66-111.52 (114.06)
Aristolochiaceae	**112.37-52.38 (81.72)**	**115.05-64.53 (91.64)**	**118.01-72.98 (96.35)**	**124.24-77.60 (101.08)**	**132.66-81.01 (108.07)**
	109.54-101.83 (105.72)	112.93-104.83 (108.84)	117.32-108.64 (113.00)	129.6-119.28 (123.70)	147.26-134.83 (140.00)
Atherospermataceae	**48.44-12.57 (29.52)**	**47.55-17.00 (30.92)**	**44.96-17.76 (30.26)**	**46.56-19.08 (31.75)**	**46.80-19.99 (32.57)**
	66.47-46.69 (57.09)	69.03-47.90 (59.25)	76.51-55.52 (65.62)	81.19-56.24 (69.09)	92.84-64.61 (79.42)
Canellaceae	**57.73-11.33 (33.42)**	**61.98-14.32 (35.75)**	**49.18-14.61 (31.55)**	**48.88-16.46 (31.35)**	**52.34-17.80 (33.75)**
	37.92-28.09 (32.67)	38.12-28.04 (33.01)	42.69-31.02 (36.17)	39.89-29.54 (34.26)	43.55-31.82 (36.23)
Calycanthaceae	**112.43-92.45 (102.58)**	**110.38-91.65 (101.15)**	**112.1-92.09 (102.39)**	**115.25-93.12 (103.41)**	**119.79-93.08 (106.70)**
	101.05-94.65 (97.86)	102.55-95.37 (99.21)	106.07-97.89 (101.80)	110.84-100.00 (105.39)	119.72-104.93 (111.93)
SL Degeneriaceae	**106.86-17.13 (59.94)**	**113.41-32.47 (78.72)**	**116.13-46.76 (90.01)**	**119.61-49.59 (92.97)**	**128.95-61.41 (99.51)**
	108.22-95.75 (101.42)	108.35-95.64 (101.41)	115.06-102.16 (107.49)	126.58-109.77 (116.49)	146.22-127.72 (134.20)
SL Eupomatiaceae	**113.69-86.00 (102.55)**	**114.19-93.92 (104.61)**	**114.77-95.17 (105.76)**	**117.93-98.12 (108.03)**	**128.33-99.33 (112.65)**
	107.5-102.9 (105.04)	108.99-102.75 (105.05)	116.35-108.55 (110.34)	128.58-117.34 (120.45)	148.39-136.13 (138.52)
SL Gomortegaceae	**93.27-24.43 (54.97)**	**99.17-35.76 (65.01)**	**98.93-36.39 (66.41)**	**102.60-36.77 (68.59)**	**102.63-36.15 (70.72)**
	105.91-88.33 (95.63)	109.36-90.77 (98.97)	115.80-100.34 (106.43)	127.29-106.28 (114.29)	145.59-122.98 (130.49)
Hernandiaceae	**94.13-35.71 (64.36)**	**93.05-44.33 (69.39)**	**93.7-48.72 (70.84)**	**95.44-50.93 (73.52)**	**100.6-55.7 (78.42)**
	100.79-80.61 (90.14)	104.82-82.65 (92.97)	108.86-85.51 (96.78)	121.19-94.86 (106.10)	137.67-107.51 (120.22)
SL Himantandraceae	**106.86-17.13 (59.94)**	**113.41-32.47 (78.72)**	**116.13-46.76 (90.01)**	**119.61-49.59 (92.97)**	**128.95-61.41 (99.51)**
	108.22-95.75 (101.42)	108.35-95.64 (101.41)	115.06-102.16 (107.49)	126.58-109.77 (116.49)	146.22-127.72 (134.20)
Lauraceae	**89.52-45.98 (66.74)**	**89.54-52.30 (70.95)**	**90.10-51.97 (71.85)**	**93.39-55.91 (75.19)**	**98.02-61.32 (79.62)**
	106.51-97.3 (100.45)	110.45-100.43 (103.73)	115.25-105.35 (109.39)	128.32-115.43 (118.92)	146.61-131.04 (135.25)
Magnoliaceae	**81.67-5.71 (33.77)**	**89.84-9.65 (41.60)**	**79.30-7.70 (37.84)**	**80.06-9.81 (38.12)**	**84.92-6.51 (41.88)**
	80.22-53.79 (66.59)	80.28-53.29 (66.68)	94.90-69.01 (79.27)	97.36-65.39 (79.34)	115.11-79.98 (94.04)
Monimiaceae	**86.82-39.51 (62.78)**	**85.10-43.65 (64.10)**	**87.24-44.65 (65.34)**	**88.65-47.28 (67.24)**	**94.16-50.99 (71.89)**
	98.91-85.1 (90.86)	103.57-88.32 (94.05)	111.79-99.03 (104.84)	123.24-102.36 (109.02)	143.04-118.67 (125.73)
Myristicaceae	**51.51-14.70 (32.60)**	**44.63-16.19 (29.39)**	**40.61-15.43 (27.15)**	**40.44-16.22 (27.20)**	**42.33-18.23 (29.00)**
	16.53-10.92 (14.12)	16.98-11.15 (14.37)	16.66-10.22 (13.76)	19.25-12.19 (16.12)	22.17-14.35 (18.04)
Piperaceae	**78.43-37.84 (58.02)**	**88.34-49.06 (67.60)**	**83.52-51.67 (67.20)**	**77.71-48.73 (70.35)**	**96.81-59.35 (77.72)**
	65.65-57.42 (61.55)	67.53-58.99 (62.98)	68.48-58.68 (63.37)	75.52-65.43 (69.81)	84.91-72.91 (77.40)
Saururaeae	**75.32-46.69 (60.02)**	**79.45-47.96 (61.96)**	**76.21-47.81 (60.86)**	**77.71-48.73 (62.23)**	**80.79-48.62 (63.90)**
	55.27-48.42 (51.76)	56.22-48.76 (52.28)	59.52-50.71 (54.36)	60.05-50.77 (54.92)	65.68-53.33 (58.13)
SL Siparunaceae	**113.52-38.48 (81.96)**	X-X (X)*	**114.54-60.66 (95.33)**	**119.17-67.27 (99.30)**	**124.92-64.04 (100.47)**
	114.46-105.81 (110.05)	118.43-109.28 (113.6)	123.78-114.75 (119.09)	137.16-125.45 (130.03)	157.67-143.07 (147.57)
Winteraceae	**65.07-12.11 (36.18)**	**58.94-14.93 (34.75)**	**56.38-16.33 (34.31)**	**58.95-17.47 (36.11)**	**56.73-19.08 (36.59)**
	45.62-33.34 (39.55)	46.27-33.76 (39.99)	55.94-39.68 (47.35)	48.40-35.62 (41.61)	52.66-38.52 (44.16)

All age estimates are in million years. The first line corresponds to BEAST estimates (boldface) and the second line to r8s (penalized likelihood) estimates. 95% credibility intervals (CI) are followed in brackets by mean estimates for BEAST analyses and best-scoring ML tree estimates for r8s analyses. Abbreviations: angio-130, angio-140, angio-150, angio-170, and angio-200 correspond to the different maximum age constraints applied to the root (130, 140, 150, 170, and 200 million years respectively). The names of taxa refer to their crown node except if SL (stem lineage) is mentioned. *The stem lineage of Siparunaceae, in the BEAST analysis with the maximum age of angiosperms set to 140 million years, did not correspond with the same crown node than in other analyses.

### Diversification analyses

**Figure 2:**
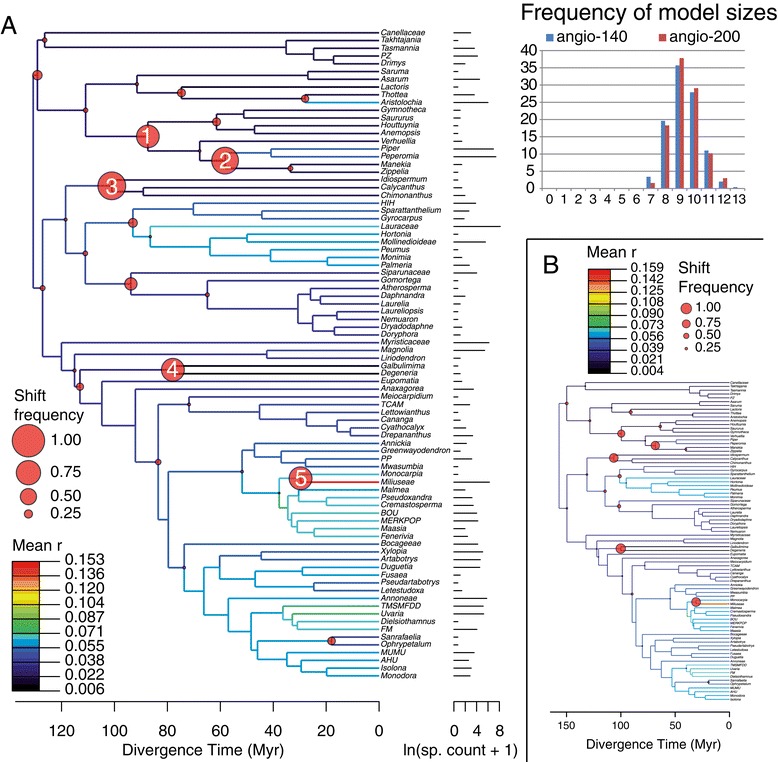
**Diversification analyses.** Results of the MEDUSA analyses obtained using 1000 posterior trees randomly sampled from the BEAST posterior: **A**, angio-140, **B**, angio-200. Topologies are the maximum clade credibility tree of 1000 randomly selected trees (phylogenetic relationships between **A** and **B** are identical). Names of terminals refer to compartments defined in the Methods sections and detailed in Additional file 5. Branch colors illustrate the mean net diversification rate (r). Red dots denote significant shifts in r, their size being proportional to their frequency among the 1000 trees tested. Numbered shifts in Figure 2A: 1, crown node of the clade of Piperaceae and Saururaceae; 2, crown node of the clade of Piperaceae excluding *Verhuellia*; 3, crown node of Calycanthaceae; 4, crown node of the clade of Himantandraceae and Degeneriaceae; 5, crown node of the clade of Miliuseae and *Monocarpia*. The diagram in the top-right corner represents the frequency of the different model sizes (number of shifts) in the 1000-model collections for the two analyses (angio-140 and angio-200). Abbreviations: sp., species; Myr, million years.

Our MEDUSA analyses on 1000 posterior BEAST trees of the angio-140 and angio-200 calibration schemes resulted in very similar scenarios for the mode of diversification within Magnoliidae (Figures 2A, B). The two maximum clade credibility trees used to summarize results are identical in terms of relationships. We identified six to 13 significant diversification rate shifts across Magnoliidae. In both cases, models including nine shifts were the most often selected among the 1000 trees sampled (Figure 2). The mean of the initial net diversification rate (speciation minus extinction) at the crown node of Magnoliidae is estimated to 0.0401 ± 0.0138 species per million years (sp.myr^−1^) in angio-140, and 0.0342 ± 0.0134 sp.myr^−1^ in angio-200. When considering shifts present in more than 50% of the trees (Figure 2), five main shifts in net diversification rates within Magnoliidae are identified (the corresponding nodes were all supported by posterior probabilities of 1.0 and are numbered on Figure 2). Three are decreases and two are increases: 1) at the crown node of the clade of Piperaceae and Saururaceae (magnitude of shift: −0.0052 ± 0.0155 sp.myr^−1^ in 70% of trees of the angio-140 analysis; −0.0032 ± 0.0130 sp.myr^−1^ in 68% of trees of the angio-200 analysis); 2) at the crown node of Piperaceae excluding *Verhuellia* (+0.0317 ± 0.0421 sp.myr^−1^ in 83% of trees of the angio-140 analysis, +0.0295 ± 0.0265 sp.myr^−1^ in 80% of trees of the angio-200 analysis); 3) at the crown node of Calycanthaceae (−0.0292 ± 0.0140 sp.myr^−1^ in 86% of trees of the angio-140 analysis, −0.0238 ± 0.0133 sp.myr^−1^ in 83% of trees of the angio-200 analysis); 4) at the crown node of the clade of *Degeneria* and *Galbulimima* (−0.0366 ± 0.0140 sp.myr^−1^ in 71% of trees of the angio-140 analysis, −0.0325 ± 0.0130 sp.myr^−1^ in 90% of trees of the angio-200 analysis); 5) at the stem node of tribe Miliuseae of Annonaceae (+0.1231 ± 0.0306 sp.myr^−1^ in 71% of trees of the angio-140 analysis, +0.1157 ± 0.0307 sp.myr^−1^ in 83% of trees of the angio-200 analysis). In addition to those five main shifts, the net diversification rate averaged over the sample of 1000 trees show a general increase both within the Laurales and within the Magnoliales (Figure 2). Our estimates of the relative extinction rate (ratio of extinction over speciation) at the crown node of Magnoliidae are 0.9000 ± 0.1533 (angio-140) and 0.8857 ± 0.1603 (angio-200) [see Additional file 4]. This rate remained relatively high (more than 0.5) in Magnoliidae except in Laurales and part of Piperales.

## Discussion

### Tempo of Magnoliidae evolution

We provide here for the first time a near complete temporal framework for the evolution of Magnoliidae above the generic level. Applying different maximum age constraints to the crown node of angiosperms highlighted the sensitivity of inferred ages estimated for the deepest nodes of Magnoliidae (Figure 1). Although previous studies have recognized a long gap in the fossil record of the angiosperm stem lineage [13,21,37], there is no indisputable argument in favor of one particular maximum age constraint for the crown node of angiosperms. Here we will systematically consider the whole range of age estimates obtained across our five maximum age calibration schemes and both molecular dating approaches used.

We restricted ourselves to conservative fossil age constraints based on phylogenetic analyses of fossil and extant taxa [36]. This led us to consider only 10 out of more than 100 described fossils putatively belonging to Magnoliidae [38]. However, our new age estimates for families and orders of Magnoliidae appear, in general, to be compatible with the putative fossil record attributed to each of these taxa. Future investigations of the phylogenetic placement of other magnoliid fossils will likely provide additional minimum age constraints, which could push back in time several young estimates supported in the present study, and decrease the size of the credibility intervals. This study further illustrates that, just as paleontological dating, molecular dating estimates are often associated with large uncertainties. The latter approach has to be seen as an attempt to reduce the range of the most likely ages for nodes constrained by the age of the fossil record securely placed and dated, and to evaluate the probability of the ages of nodes for which there is no direct fossil record. Because all the ambiguity of our current knowledge has to be taken into account, the molecular dating approach cannot provide exact secure ages, except for exceptionally fossil-rich clades.

Based on this most reliable knowledge in the field of paleobotany of Magnoliidae [36] and chrono-stratigraphy [39], combined with a comprehensive sample of taxa and molecular markers, our data support an origin of extant lineages of Magnoliidae (crown node of the group) during the Jurassic or Lower Cretaceous between 178.78 and 126.82 Myr. This result tend to be older than previously suggested [12,15,21,22,37,40-42]. For example, the angiosperm-wide study of Bell et al. [37], in which 33 magnoliid genera were sampled, estimated the crown node of Magnoliidae to be between 138 and 108 Myr. In another, more recent angiosperm-wide analysis sampling 34 species of Magnoliidae and using a PL approach, Zanne et al. [42] estimated the age of the crown node of Magnoliidae to be 147 Myr without uncertainty associated. Our new estimates for the crown-group age of orders also tend to be older than previously inferred [13,15,16,22,37] (Figure 1). However, some studies have supported much older ages for these nodes [13,14,21,43]. At the familial level, in addition to providing the first estimates for the ages of crown-group Canellaceae (52.34-11.33 Myr), Saururaceae (80.79-46.69 Myr), and Winteraceae (56.73-12.11 Myr; Table 1), the crown nodes of families are also generally dated to an older range of dates than previously estimated [16-20,26,44], with the exception of Atherospermataceae (Laurales) for which we found a younger age than previously suggested [45]. The large amount of combinations of parameters influencing age estimates limits straightforward explanations of these differences with previous studies (e.g., topology used [46]; fossil species and priors used to define and model age constraints [47]; molecular dating method [13]). Despite variation among the ages obtained for the crown ages of families, our results confirm that Magnoliidae diversified into morphologically distinct clades (now identified as families) by the end of the Cretaceous (66 Myr, Figure 1), but probably earlier than thought before [16-20,26,44]. This provides arguments in favor of an earlier diversification of angiosperms altogether, with the presence of more flowering plant lineages in the Cretaceous than previously thought.

Finally, taking into account the whole range of age estimates obtained across our five different calibration schemes, leads to long ranges of age estimates that are compatible with biogeographic scenarios previously suggested in Magnoliidae [17,26,44,45,48-52]. However, viewed in isolation, the different assumptions about the maximum age of angiosperms lead to alternative time scales for the evolutionary history of the families of Magnoliidae. Therefore, biogeographic scenarios will need to be reevaluated in the context of explicit assumptions on the age of the angiosperms.

### Mode of diversification of Magnoliidae

The new timetree obtained for Magnoliidae in this study allowed us to detect an average of nine significant diversification rate shifts across lineages, implying that diversification has not been a homogeneous process throughout the history of the clade (Figure 2). Explaining the present day diversity of species-rich clades has generally required several diversification rate shifts, but previous studies using the same statistical approach in other large angiosperm clades found, in general, fewer shifts than in the present analysis. Within eudicots, Beaulieu and Donoghue [53] detected six shifts in Campanulidae (35,000 spp.), Koenen et al. [54] detected nine shifts in Fabaceae (19,500 spp.), Xi et al. [55] found five significant shifts in Malpighiales (16,000 spp.), and Arakaki et al. [8] found seven shifts in Cactaceae (1850 spp.). Within monocots, Escudero and Hipp [56] supported three shifts in the family Cyperaceae (5480 spp.) while Baker and Couvreur [57] identified up to 13 shifts in palms (2500 spp.). Despite the dependence of the number of shifts detected on the taxonomic level of tips in the chronograms used (MEDUSA cannot detect shifts within supraspecific terminal compartments), it seems that Magnoliidae experienced a high variation in diversification rates. Because of their longer evolutionary history in comparison with these other clades and because their fossil record supports a global distribution during the Cretaceous and the Paleogene [38], Magnoliidae as a whole may have been affected by more events potentially influencing the speciation and extinction rates (e.g., climatic shifts [58]; variation in available area [10]; variation in geographic distribution [59]; dynamic of diversification of associated pollinators [60]).

Establishing the causes affecting diversification-rate variation can be difficult. There is a large diversity of intrinsic and extrinsic traits known to influence speciation and extinction [10], all of them being potentially correlated to each other [61]. The influence of a trait on the birth and death of species is dependent on other taxa, other traits of the same organism, and its physical environment [62], implying that a trait could have different effects on different clades. Finally, the actual shift of diversification may happen several nodes after the evolution of an influencing trait. One explanation could be that an isolated trait will influence diversification in combination with other characters, the effect appearing when the entire set of traits needed is present [53]. For these reasons, the investigation of the correlation between traits and diversification rates will need to take into account as many potential factors as possible [63].

The older estimates of the ages in the “angio-200” hypothesis led to slightly lower absolute rates of diversification than in the “angio-140” hypothesis, as expected. Because the general pattern (number and position of shifts) is the same in both analyses, in the present paragraph we refer only to rates of the angio-140 analysis. The background net diversification rate obtained in the tree of Magnoliidae (0.0401 ± 0.0138 sp.myr^−1^) is comparable to those found in angiosperms [14,24]. In the present study, the lowest net diversification rates, resulting from shifts 3 and 4 (Figure 2), were found in the crown group of Calycanthaceae (0.0175 ± 0.0066 sp.myr^−1^), and in the branches of Degeneriaceae and Himantandraceae (0.0057 ± 0.0088 sp.myr^−1^). These three families share distinctive floral features not or seldom found in other Magnoliidae groups, such as the presence of inner staminodes that cover the stigma at the end of the female phase [64], and spiral phyllotaxy of all floral organs [65,66]. However, it is difficult to link these two characters with mechanisms sustaining a low diversification rate (influence on speciation and/or extinction). Within Calycanthaceae, the low rate of net diversification is associated with a low speciation rate for the entire clade (0.0182 sp.myr^−1^) and near-zero extinction (0.0007 sp.myr^−1^). The absence of extinction would seem contradictory with the presence of several fossils in the Cretaceous [17] and could be an artefact of the method which does not take into account the extinct diversity (see below). However, because the phylogenetic position of these fossils within the family has not been tested (except for *Virginianthus calycanthoides* [67]), it is difficult to draw conclusions about extinction within the family. The pollination systems of Calycanthaceae involve trapping of a large variety of beetles and thrips [68,69]. In comparison with specific pollination systems involving a unique relationship for the two partners, this generalist interaction is not favorable for genetic isolation promoting diversification [70]. In addition, Calycanthaceae have a temperate distribution, except for the monospecific genus *Idiospermum* growing in a restricted area in north-east Australia. The low ecological limits on species richness in temperate areas [10] could provide an explanation for the low diversification of the lineage leading to the genera *Calycanthus* and *Chimonanthus*. On the other hand, the apparent lack of diversification of the lineage leading to *Idiospermum* could be explained by different causes. Individuals of this species are long-living trees [71] involving low fixation rates in diverging populations. The genus is restricted to the very wet humid tropical lowland rainforests of Australia [72]. *Idiospermum* presents one of the heaviest seeds among Australian plants and is therefore probably not dispersed by animals, limiting its capacity of dispersion [71,73]. If this low capacity of dispersion is ancestral in the lineage leading to the extant species of the genus, it restrained the number of available areas promoting diversification [10]. This latter explanation might be generalized for all Calycanthaceae as the seeds of the family contain secondary metabolites that are toxic at least for mammals [74].

The highest diversification rate inferred in this study was found on the branch leading to the terminal compartment Miliuseae in subfamily Malmeoideae of Annonaceae (0.1527 ± 0.0548 sp.myr^−1^). A shift of +0.1231 ± 0.0306 sp.myr^−1^ at the node sustaining the branch of this clade was present in 71% of the trees tested and the associated rate of speciation on the branch is on average equal to 0.4558 sp.myr^−1^. Couvreur et al. [26] found that major clades in Annonaceae have undergone different rates of diversification, with subfamily Malmeoideae (previously the Short-Branch Clade) having significantly higher rates than subfamily Annonoideae (previously the Long-Branch Clade). However, analyses of family-level lineage-through-time plots (LTTs) did not detect significant shits in diversification rates during most of the evolutionary history of Annonaceae [26]. Erkens et al. [25], using a tree with fewer taxa and two alternative approaches to detect rate shifts, identified up to three diversification rate shifts in Annonaceae, including the one we detected here at the crown of Miliuseae. Reasons for an increase in diversification within Miliuseae are not yet well known but could be related to founder effects after dispersal into South-East Asia or distinctive pollen characters [25].

Canellales and Piperales have, on average, higher speciation rates than Magnoliales and Laurales. Within Piperales, this may be explained by widespread herbaceous habits, resulting in faster life history (shorter generation time) and higher fixation rates in divergent populations [75]. In addition, within this order, Aristolochiaceae typically have zygomorphic flowers, which has been shown to have a positive impact on diversification [70]. However, Canellales and Piperales also share the highest extinction rates, making Laurales and Magnoliales the most productive orders in terms of net diversification. Differences in extinction rates are difficult to explain. For instance, there are no major differences in pollination systems among the four orders [64], and their current geographic distributions are similar [76]. However, Doyle and Endress [77] found several synapomorphies for the clade of Laurales and Magnoliales, specifically the presence of more than two whorls of stamens and more than one whorl or series of carpels. These characters might have played a role in relation to pollination, but the underlying mechanisms remain unknown.

Finally, even though the MEDUSA approach represents a significant conceptual improvement over previous models, it still requires several important assumptions that may have influenced our results. First, MEDUSA does not implement diversification models in which the extinction rate can be higher than speciation rates. Second, the rates are assumed to be constant in each part of the tree, and only abrupt variations are evaluated, probably not in accordance with the biological reality in which rates could gradually fluctuate. Morlon et al., [78] recently developed an approach specifically to relax these two assumptions, but it is not implemented yet to detect shifts automatically nor to analyze higher-level phylogenies with terminally unresolved clades. In addition, Rabosky [79] recently developed a new Bayesian framework to detect multiple diversification processes in a tree while allowing the speciation rate to vary continuously through time, but it is not available yet for a set of multiple trees and therefore cannot take phylogenetic and dating uncertainty into account. Last, because the fossil record is not taken into account in the diversification rate analyses, very low rates of speciation and extremely low extinction rates are always allocated to the old lineages with few species (long branches in chronograms; [1]), which have probably been more diverse in the past (e.g., *Idiospermum*). New research efforts are currently being made in this area [80,81]. Therefore, our understanding of Magnoliidae diversification may further improve in the future when more realistic methods and more complete phylogenies are available.

## Conclusions

The present study suggests that Magnoliidae began to diversify somewhere between the Toarcian (Early Jurassic) and the Barremian (Early Cretaceous), or 178.78-126.82 Myr ago. Several key nodes within Magnoliidae are dated here for the first time. In general, our age estimates suggest a possibly older diversification of the group than previously inferred. The rich fossil record of Magnoliidae may eventually provide additional calibration points to refine the time scale proposed here. However, considerable work remains to be done to securely relate this extinct diversity to the extant one, a task for which an integrated morphological dataset will be essential. The tempo and mode of diversification within this clade appears to be characterized by several increases and decreases of diversification rates, suggesting that Magnoliidae have not undergone constant diversification but have instead been shaped by alternative, yet to be determined evolutionary processes. Our new dated trees provide a solid basis for future biogeographical studies and robust statistical tests of correlation among intrinsic traits, extrinsic factors, and diversification rates within Magnoliidae.

## Methods

### Molecular dataset

In order to conduct the molecular dating analyses we used the same 12-marker molecular dataset as [33]. This matrix includes 12 coding and non-coding markers from the three genomes: *atpB*, *matK*, *trnL* intron, *trnL-trnF* spacer, *ndhF*, *rbcL* from the chloroplast; *atp1*, *matR*, *mtSSU*, *mtLSU* from the mitochondrion; and 18S rDNA and 26S rDNA from the nucleus. In this dataset, we used an exemplar approach, in which each genus was represented by one species. The problematic parasitic family Hydnoraceae was excluded because its exact position within Piperales remains difficult to assess [16,33]. All remaining 19 families of Magnoliidae are represented, and more than 75% of the genera were sampled (198 genera out of 262). In addition, we included 23 outgroup taxa sampled from early-diverging angiosperms (Amborellales, Nymphaeales, Austrobaileyales, Chloranthales) as well as eudicots and monocots [see Additional file 3].

### Molecular dating analyses

#### Calibration scheme

**Figure 3:**
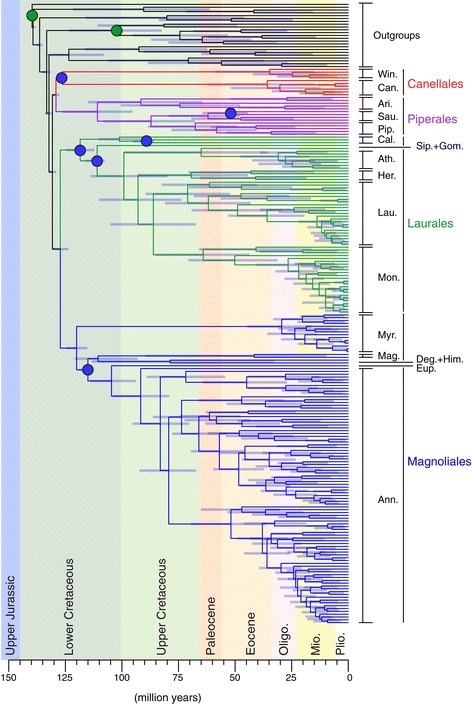
**Chronogram of the angio-140 analysis obtained with BEAST.** Maximum clade credibility tree obtained with BEAST and the maximum age constraint for the crown node of angiosperms set to 140 million years. Node bars are 95% credibility intervals. Blue dots symbolize minimum age constraints, and green dots the maximum age constraints applied to crown eudicots and crown angiosperms. The geologic time scale follows Gradstein et al. [39]. Abbreviations: Oligo., Oligocene; Mio., Miocene; Plio., Pliocene; Win., Winteraceae; Can., Canellaceae; Ari., Aristolochiaceae (incl. *Lactoris*); Sau., Saururaceae; Pip., Piperaceae; Cal., Calycanthaceae; Ath., Atherospermataceae; Her., Hernandiaceae; Lau., Lauraceae; Mon., Monimiaceae; Myr., Myristicaceae; Deg., Degeneriaceae; Him., Himantandraceae; Mag., Magnoliaceae; Eup., Eupomatiaceae; Ann., Annonaceae.

All the geological ages presented in this study follow the revised Geological Time Scale of Gradstein et al. [39]. To calibrate the molecular phylogeny we use the calibration scheme proposed by Massoni et al. [36]. This scheme consists of 10 fossils reviewed for both their phylogenetic positions (based exclusively on explicit phylogenetic analyses) and their absolute age based on the latest stratigraphic and geochronological literature (Table 2), resulting in solid minimum age constraints on six nodes of our tree (Figure 3). In addition to these six minimum age constraints, we used two maximum ages. First, the crown node of eudicots was set to a conservative maximum of 126.7 Myr (126.3 ± 0.4 Myr), based on the first appearance of tricolpate pollen grains near the late Barremian-early Aptian boundary [82,83]. This maximum age constraint is justified by the fact that tricolpate pollen is a synapomorphy of eudicots and that the absence of such pollen in well sampled earlier sediments worldwide is well documented. It has been used extensively in previous molecular dating analyses of angiosperms as a whole [14] and of clades nested within eudicots [47]. Second, the crown node of angiosperms was set to five different maximum ages in order to test the sensitivity of the Magnoliidae divergence time scale to this temporal constraint. The age of the crown node of angiosperms is a matter of debate [84]. Several pre-Cretaceous fossils for the angiosperms have been described but their ages and phylogenetic affinities with the angiosperms (stem relatives of angiosperms, crown members of angiosperms, or gymnosperms) are controversial [84]. The oldest fossil pollen confirmed to be angiospermous (either crown or stem), based on columellar exine structure, is dated to the Hauterivian or Valanginian (i.e. 139.4-133.9 Myr [84]). On the other hand, molecular dating studies have generally converged to an age for the crown node of angiosperms between 140 and 200 Myr [12,15,22,37,40,41] with few of them inferring older ages [13,43]. In order to take into account this uncertainty on the crown age of angiosperms, we used five different age constraints: 130 Myr (angio-130 analyses), 140 Myr (angio-140 analyses), 150 Myr (angio-150 analyses), 170 Myr (angio-170 analyses) and 200 Myr (angio-200 analyses) for the maximum age constraint applied to the crown node of angiosperms in all molecular dating analyses.

**Table 2:** **Fossil species used to define the calibration scheme presented in Massoni et al.** [36]

**Fossil**	**Age (Myr)**	**Node**
*Archaeanthus linnenbergeri*	96.5	crown-group Magnoliineae
*Cohongarootonia hispida*	107.7*	crown-group core Laurales
*Endressinia brasiliana*	112.6*	crown-group Magnoliineae
*Jerseyanthus calycanthoides*	85.8*	crown-group Calycanthoideae
*Lovellea wintonensis*	100.1	crown-group Laurales
*Mauldinia mirabilis*	95.5	crown-group core Laurales
*Saururus tuckerae*	44.3*	stem node of extant *Saururus*
*Schenkeriphyllum glanduliferum*	112.6*	crown-group Magnoliineae
*Virginianthus calycanthoides*	107.7*	crown-group Laurales
*Walkeripollis gabonensis*	125.9*	crown-group Canellales

*Date effectively used as calibration (some fossils with different ages may calibrate the same node, in this case we use the oldest fossil). Abbreviation: Myr, million years.

#### Divergence time estimation

##### Uncorrelated Lognormal Clock (UCLN) analyses

To evaluate divergence times within Magnoliidae while taking into account phylogenetic uncertainty, we used BEAST v1.7.5 [85] without fixing the tree. In all BEAST analyses, we partitioned our molecular dataset according to the 12 markers (as in Massoni et al. [33]). We used MrModeltest 2.3 [86] to evaluate the best fitted model for each partition. For all partitions, GTR + Gamma was selected as the most appropriate model according to the Akaike Information Criterion, and the evaluation of invariant sites (I) was recommended for all, except for the plastid spacer trnL-trnF. A Birth-Death incomplete sampling prior was specified for the trees [87], and rate heterogeneity was modeled using the UCLN relaxed clock of Drummond et al. [88] with a uniform prior for the mean of the branch rate set between 0 and 1E100. All age constraints were applied using uniform priors (hard minimum and maximum ages) because of the difficulty of parameterizing non-uniform priors, which may involve subjectivity [47]. These uniform distributions were bounded either by the age of the fossil (Table 2) and 1E100 Myr for minimum age constraints, or by the appropriate maximum age (see above) and 0 Myr for maximum age constraints. Because in BEAST the construction of the calibrated tree prior is a multiplicative construction involving the multiplication of the calibration densities (specified by the user) with the tree prior, the effective calibration priors used in analyses can differ from the specified priors [89]. This can cause the effective prior to violate the desired paleontological constraints [90]. We conducted all BEAST analyses without the data in order to estimate the mismatch between the specified and effective calibration priors, but also the effect of data on posterior distributions. Because of initial likelihood problems at the start of the analyses we used a starting tree obtained from the Bayesian analysis of Massoni et al. [33] and rendered ultrametric using Penalized Likelihood (PL) implemented in r8s v1.8 [91]. Because of rounding to a limited number of decimals, branch times in this starting tree summed up to slightly older node ages than its original calibrations. Thus, in order to use this starting tree, we had to apply slightly older (+0.1 Myr) maximum age constraints (126.8, 130.1, 140.1, 150.1, 170.1, and 200.1 Myr). For all analyses, we ran four independent chains of a Markov Chain Monte Carlo procedure for 100 million generations each, sampling parameters and trees every 1000 generations [see Additional file 1]. All analyses were performed on the CIPRES cluster [92]. We evaluated the size of the burnin phase for each run using Tracer v1.5 [93]. The post-burnin posteriors of the four runs of each analysis were then combined using LogCombiner v1.7.5. Because of computational limitations in relation to the large size of generated files, we re-sampled trees and parameters every 10,000 or 15,000 generations [see Additional file 1]. We then used TreeAnnotator v1.7 to select the maximum clade credibility (MCC) tree of each analysis.

##### Penalized Likelihood (PL) analyses

We also conducted PL analyses for each set of age constraints using r8s v1.8 [91]. The PL relaxed clock assumes some degree of autocorrelation of molecular substitution rates between a parent and its immediate descendants [94]. Although autocorrelated relaxed clock models are also available in Bayesian frameworks, our rationale for using r8s was to test the sensitivity of our age estimates to a fundamentally different approach to molecular dating. In all analyses, we used the best scoring maximum likelihood (ML) phylogram of Massoni et al. [33] obtained with the 12-marker RAxML analysis excluding *Hydnora*. In order to determine the optimal level of autocorrelation across the tree (smoothing parameter), we conducted cross validation procedures testing 17 different values of smoothing in a range between 0.1 and 10,000,000 for each calibration scheme, using the penalty additive function. For angio-130, angio-140, angio-170 and angio-200 the optimal values were 10, and for angio-150 it was 3.2. In order to provide confidence intervals on age estimates with PL, we conducted non-parametric maximum likelihood bootstrapping of 1000 replicates on the original dataset of Massoni et al. [33] using RAxML v7.3.2 [95] while fixing the tree topology to the best scoring ML tree obtained in this previous study. As RAxML requires the application of the same model to all partitions, we used the GTR + GAMMA + I model for each partition. As mentioned above, this model was selected for all partitions of the present dataset except for the plastid spacer *trnL-trnF* for which the invariant site was not recommended. Within the resulting 1000-tree collection, only branch lengths varied. We reconstructed the chronograms for both the best ML tree and the 1000 bootstrapped phylograms using the optimal smoothing parameter obtained for the best ML tree and the TN algorithm of r8s. All results were summarized using the software TreeAnnotator v1.7 with the 1000 PL trees as the input file and the ML-PL tree as the target tree.

### Diversity dynamics

We used the MEDUSA approach of Alfaro et al. [1] to test for significant diversification rate shifts in an incompletely sampled phylogeny where each terminal is assigned the total number of extant species it represents. MEDUSA detects, using a stepwise AIC approach, significant shifts of diversification rates across a given phylogeny by evaluating the fit of different pairwise birth-death models. In order to select the best model we used the AICc criterion, a birth-death model of diversification, and we allowed the placement of shifts either on stem or on crown nodes. To assess the sensitivity of MEDUSA to age and phylogenetic uncertainty, we conducted the analyses on two collections of 1000 posterior BEAST trees randomly sampled from the post-burnin phase, corresponding to calibration scenarios angio-140 and angio-200. Prior to the analyses, each randomly sampled chronogram was transformed in two ways, using functions in the ape package of R [96]. First, all outgroups of Magnoliidae were pruned. Second, the chronograms were simplified by pruning selected terminal taxa so that each remaining terminal taxon would represent a monophyletic compartment with known extant diversity (see below). All analyses were performed in R using the multiMEDUSA procedure from package MEDUSA v0.93 4.33 available on the website authored by Joseph W. Brown (https://github.com/josephwb/turboMEDUSA).

#### Extant taxonomic richness data

In order to assign the entire species richness of Magnoliidae to the tips of the phylogeny, it was necessary in several instances to merge tips into a single terminal to represent larger monophyletic groups hereafter referred to as compartments. We proceeded in two steps. First, we maintained genera as tips or created larger compartments, depending on the monophyletic status of genera as tested in previous phylogenetic studies: if the literature supported the monophyly of a genus, this taxon was used as a compartment; if the monophyly of a genus had never been tested before, a compartment including this genus and its sister group was defined (or a larger compartment if the neighborhood relationships were not well supported); if the monophyly of a genus had been challenged by previous studies, we defined a larger compartment to include this genus and all other genera potentially involved in the paraphyly or polyphyly. After this first step, we modified our compartmentalization scheme to take into account the species numbers of genera not sampled in our trees (less than 25% of the total number of accepted genera). If there was enough information in the literature to support an accurate placement (phylogenetic or apomorphy-based) of a missing genus, we added its number of species to that of an earlier defined compartment (generic or supra-generic). In some cases, the missing genus could not be precisely placed within a larger clade of two or more previously defined compartments. As a guideline, we accepted to merge these compartments into a larger one if the number of species would represent more than three percent of the total number of species in the resulting compartment. Otherwise, the missing diversity was ignored in order to maintain enough compartments for conducting a meaningful analysis. In all cases, we defined these supra-generic compartments in such a way that they were present in all 1000 BEAST trees used to conduct the MEDUSA analyses (posterior probabilities [PP] equal to 100%). The resulting compartmentalization scheme consisted in 85 terminal taxa [see Additional file 5]. In total, 31 species (16 genera) were ignored due to unknown phylogenetic placement, representing ca. 0.3% of the 10,209 species of Magnoliidae. For 15 genera (out of 262) the number of species was not clearly indicated in previous publications in which case the Plant List (http://www.theplantlist.org/) was used to estimate the number of extant species currently accepted in the genus. The definition of supra-generic clades and the incorporation of the missing diversity are justified in detail in Additional file 5.

## Availability of supporting data

Nexus files including the MCC trees of the BEAST analyses and the PL trees of the r8s analyses (annotated with the 95% credibility intervals of age estimates) are available in Dryad Digital repository, http://dx.doi.org/10.5061/dryad.ct231 [97].

## Additional files

Additional file 1:
**Information about BEAST analyses.**
Additional file 2:
**Illustration of the compartments for the diversification analyses.** Tree modified from the maximum clade credibility tree of the BEAST angio-140 analysis (branch times contracted in some clades for graphical purposes). Numbers at nodes are posterior probability values, which are compatible with all other BEAST analyses. Green terminals are the compartments used for the diversification analysis. Braces highlight clades in our chronograms collapsed as terminals. We used the abbreviation ‘incl.’ to indicate where the species richness of unsampled genera has been counted, and ‘excl.’ where it was ignored. Additional file 3:
**Maximum clade credibility trees.** NEXUS file containing the maximum clade credibility trees of BEAST analyses annotated with post-burnin posteriors. Additional file 4:
**Relative extinction rates.** Same figure as Figure 2, but illustrating variation in relative extinction rates. Results of the MEDUSA analyses obtained using 1000 chronograms randomly sampled from the BEAST posterior: A, angio-140, B, angio-200. The topologies are the maximum clade credibility trees of the 1000 trees used. Names of leaves refer to terminal compartments defined to conduct this analysis. Branch colors illustrate the mean relative rate of extinction (epsilon). Red dots denote significant shifts in net diversification rate (r), their size being proportional to their frequency among the 1000 trees tested. Abbreviation: Myr, million years. Additional file 5:
**Definition of compartments for diversification analyses.** This appendix provides tables justifying the monophyly and the number of species within compartments. In addition, a text provides all justifications for the definition of supra-generic compartments, for assignment of species richness of genera not sampled in our chronograms, and for occasionally ignoring such missing genera.
