# Supplementary material for: Five major shifts of diversification through the long evolutionary history of Magnoliidae (angiosperms)
Source: BMC Evol Biol. 2015 Mar 18;15:49. doi: 10.1186/s12862-015-0320-6 (PMC4377182; doi:10.1186/s12862-015-0320-6)
Supplement: Additional file 1: — Information about BEAST analyses. [file 12862_2015_320_MOESM1_ESM.pdf]

Additional file 1. Information about BEAST analyses.

Angio 130

Run	Burnin in million generations	Frequency used to summarize results from tree files in number of generations
1	20	10,000
2	20	
3	30	
4	20	

Statistic of the combined four runs	Mean	ESS
posterior	-174300	257.113
prior	-2782.104	94.536
likelihood	-171500	5229.41
treeModel.rootHeight	130.004	4857.401
tmrca(All)	130.004	4857.401
tmrca(Caly_Chimo)	90.215	302.935
tmrca(Canellales)	125.998	4185.694
tmrca(core_Laurales)	111.034	533.718
tmrca(Laurales)	117.38	177.99
tmrca(Saur_Gymno)	50.883	370.21
tmrca(eudicots)	98.925	89.127
tmrca(root)	129.909	2334.086
tmrca(Magnoliinae)	114.765	306.681
birthDeath.meanGrowthRate	0.02897	467.895
birthDeath.relativeDeathRate	0.37	2530.773
birthDeath.sampleProbability	0.554	780.924
18s.ac	0.186	14482.993
18s.ag	0.346	8928.639
18s.at	0.213	15496.73
18s.cg	0.104	14130.476
18s.gt	0.141	14770.279
18s.frequencies1	0.242	9620.275
18s.frequencies2	0.232	10070.495
18s.frequencies3	0.267	10101.218
18s.frequencies4	0.259	9176.812
18s.alpha	0.325	10463.249
18s.pInv	0.484	10045.543
26s.ac	0.09252	10485.793
26s.ag	0.385	6037.644
26s.at	0.186	13868.77
26s.cg	0.05935	12166.208
26s.gt	0.127	16490.447
26s.frequencies1	0.263	6324.192
26s.frequencies2	0.236	8272.734
26s.frequencies3	0.309	8451.109

26s.frequencies4	0.192	6077.299
26s.alpha	0.601	5272.898
26s.pInv	0.215	5229.054
atp1.ac	0.761	11982.543
atp1.ag	0.818	7595.59
atp1.at	0.779	10527.547
atp1.cg	0.301	13747.138
atp1.gt	0.389	10529.382
atp1.frequencies1	0.28	8820.197
atp1.frequencies2	0.206	10259.053
atp1.frequencies3	0.243	10814.179
atp1.frequencies4	0.27	10290.169
atp1.alpha	0.293	6873.677
atp1.pInv	0.415	7217.586
atpb.ac	0.233	13900.691
atpb.ag	0.795	8904.992
atpb.at	0.07539	16490.457
atpb.cg	0.147	19852.628
atpb.gt	0.122	15632.692
atpb.frequencies1	0.292	8688.414
atpb.frequencies2	0.203	9775.191
atpb.frequencies3	0.235	9714.637
atpb.frequencies4	0.27	10018.052
atpb.alpha	0.502	4565.687
atpb.pInv	0.308	4638.021
matk.ac	0.454	4990.18
matk.ag	1.048	3448.828
matk.at	0.06806	9566.318
matk.cg	0.306	7465.344
matk.gt	0.308	7168.904
matk.frequencies1	0.29	3833.406
matk.frequencies2	0.151	6634.114
matk.frequencies3	0.153	5112.047
matk.frequencies4	0.406	3420.699
matk.alpha	1.153	9474.688
matk.pInv	0.137	8939.23
matr.ac	0.96	9138.396
matr.ag	0.63	6834.057
matr.at	0.724	10829.659
matr.cg	0.249	10356.765
matr.gt	0.84	9006.445
matr.frequencies1	0.263	9951.457
matr.frequencies2	0.266	10389.067
matr.frequencies3	0.254	10418.168
matr.frequencies4	0.217	9177.335
matr.alpha	0.355	8620.931
matr.pInv	0.0963	9451.468
mtlsu.ac	1.246	9261.767
mtlsu.ag	0.624	6784.51
mtlsu.at	0.862	8778.783

mtlsu.cg	0.475	9666.286
mtlsu.gt	1.039	8460.06
mtlsu.frequencies1	0.259	11239.304
mtlsu.frequencies2	0.232	10677.283
mtlsu.frequencies3	0.284	10476.503
mtlsu.frequencies4	0.225	10889.666
mtlsu.alpha	0.291	17112.972
mtlsu.pInv	0.465	17954.386
mtssu.ac	0.798	8175.174
mtssu.ag	0.437	6170.214
mtssu.at	0.63	8933.795
mtssu.cg	0.291	10343.336
mtssu.gt	0.881	8903.737
mtssu.frequencies1	0.252	10802.461
mtssu.frequencies2	0.232	10405.572
mtssu.frequencies3	0.308	10384.931
mtssu.frequencies4	0.207	9824.417
mtssu.alpha	0.292	12119.301
mtssu.pInv	0.42	12307.476
ndhf.ac	0.366	6419.006
ndhf.ag	1.225	3228.123
ndhf.at	0.09258	8218.063
ndhf.cg	0.433	8318.368
ndhf.gt	0.27	6013.169
ndhf.frequencies1	0.301	4168.022
ndhf.frequencies2	0.152	4973.539
ndhf.frequencies3	0.143	4610.634
ndhf.frequencies4	0.404	3851.226
ndhf.alpha	0.831	5951.163
ndhf.pInv	0.331	5971.007
rbcl.ac	0.362	7477.277
rbcl.ag	0.799	4030.238
rbcl.at	0.124	7166.501
rbcl.cg	0.27	9137.621
rbcl.gt	0.212	6277.846
rbcl.frequencies1	0.28	5627.507
rbcl.frequencies2	0.182	4752.235
rbcl.frequencies3	0.235	5893.579
rbcl.frequencies4	0.302	6208.969
rbcl.alpha	0.609	13726.135
rbcl.pInv	0.442	13448.942
trnlfspr.ac	0.46	3159.224
trnlfspr.ag	0.851	2175.532
trnlfspr.at	0.196	3924.115
trnlfspr.cg	0.364	5145.905
trnlfspr.gt	0.369	5098.541
trnlfspr.frequencies1	0.328	2609.098
trnlfspr.frequencies2	0.162	4142.016
trnlfspr.frequencies3	0.169	4378.165
trnlfspr.frequencies4	0.341	2563.793

trnlfspr.alpha	0.878	18361.011
trnli.ac	0.374	7291.74
trnli.ag	0.774	4388.265
trnli.at	0.14	9388.919
trnli.cg	0.307	10072.921
trnli.gt	0.467	7167.096
trnli.frequencies1	0.404	4917.58
trnli.frequencies2	0.146	6363.745
trnli.frequencies3	0.177	6098.677
trnli.frequencies4	0.272	5198.855
trnli.alpha	1.91	15444.299
trnli.pInv	0.206	16454.651
ucld.mean	0.0007218	109.823
ucld.stdev	1.1	166.855
meanRate	0.0004935	98.86
coefficientOfVariation	1.847	171.794
covariance	0.373	208.732
18s.treeLikelihood	-9412.229	9712.086
26s.treeLikelihood	-24135.457	6962.896
atp1.treeLikelihood	-6078.395	6705.179
atpb.treeLikelihood	-11797.842	6241.554
matk.treeLikelihood	-19840.387	1862.214
matr.treeLikelihood	-9752.98	9321.083
mtlsu.treeLikelihood	-11105.888	10964.323
mtssu.treeLikelihood	-7532.841	6037.265
ndhf.treeLikelihood	-32005.451	7228.671
rbcl.treeLikelihood	-18832.332	468.293
trnlfspr.treeLikelihood	-12387.86	996.978
trnli.treeLikelihood	-8590.177	971.976
speciation	-1011.668	92.823

Angio 140

Run	Burnin in million generations	Frequency used to summarize results from tree files in number of generations
1	10	15,000
2	10	
3	10	
4	10	

Statistic of the combined four runs	Mean	ESS
posterior	-174300	583
prior	-2796.208	141.663
likelihood	-171500	1553.539
treeModel.rootHeight	139.696	3059.052
tmrca(All)	139.696	3059.052
tmrca(Caly_Chimo)	89.122	582.891
tmrca(Canellales)	126.377	1525.936

tmrca(core_Laurales)	110.959	952.553
tmrca(Laurales)	118.45	231.41
tmrca(Saur_Gymno)	51.467	554.004
tmrca(eudicots)	102.684	160.112
tmrca(root)	139.298	1534.184
tmrca(Magnoliinae)	115.027	730.264
birthDeath.meanGrowthRate	0.02827	866.078
birthDeath.relativeDeathRate	0.351	5633.284
birthDeath.sampleProbability	0.525	1228.168
18s.ac	0.186	17994.01
18s.ag	0.346	11169.648
18s.at	0.213	17787.622
18s.cg	0.104	17745.457
18s.gt	0.14	18158.875
18s.frequencies1	0.242	10968.229
18s.frequencies2	0.232	11913.97
18s.frequencies3	0.267	11880.435
18s.frequencies4	0.26	10914.187
18s.alpha	0.325	12974.625
18s.pInv	0.484	12359.253
26s.ac	0.09265	12384.905
26s.ag	0.385	6088.685
26s.at	0.186	14492.506
26s.cg	0.05938	13045.874
26s.gt	0.126	19748.612
26s.frequencies1	0.263	7349.816
26s.frequencies2	0.236	8755.217
26s.frequencies3	0.309	10427.737
26s.frequencies4	0.192	6633.56
26s.alpha	0.602	6939.759
26s.pInv	0.216	6773.076
atp1.ac	0.759	14229.626
atp1.ag	0.814	8979.292
atp1.at	0.776	12709.919
atp1.cg	0.3	16040.39
atp1.gt	0.387	11967.494
atp1.frequencies1	0.28	11012.798
atp1.frequencies2	0.206	12435.948
atp1.frequencies3	0.243	12716.644
atp1.frequencies4	0.27	11672.399
atp1.alpha	0.293	8734.171
atp1.pInv	0.415	8443.193
atpb.ac	0.233	14830.1
atpb.ag	0.796	9647.35
atpb.at	0.07535	21066.139
atpb.cg	0.148	22171.179
atpb.gt	0.122	19367.62
atpb.frequencies1	0.293	10597.006
atpb.frequencies2	0.202	11600.661
atpb.frequencies3	0.235	11378.865

atpb.frequencies4	0.27	10691.856
atpb.alpha	0.506	5112.562
atpb.pInv	0.31	5060.469
matk.ac	0.455	6339.678
matk.ag	1.051	4492.098
matk.at	0.06819	11774.317
matk.cg	0.307	9689.776
matk.gt	0.309	8691.134
matk.frequencies1	0.29	4829.837
matk.frequencies2	0.151	7620.338
matk.frequencies3	0.153	6282.893
matk.frequencies4	0.406	4617.881
matk.alpha	1.148	10327.379
matk.pInv	0.135	10653.845
matr.ac	0.957	10365.761
matr.ag	0.627	8383.35
matr.at	0.722	12866.265
matr.cg	0.248	12800.082
matr.gt	0.837	11247.167
matr.frequencies1	0.263	10802.139
matr.frequencies2	0.266	11955.2
matr.frequencies3	0.254	12042.599
matr.frequencies4	0.217	11274.521
matr.alpha	0.354	12614.105
matr.pInv	0.09525	11801.249
mtlsu.ac	1.247	12205.828
mtlsu.ag	0.623	8690.11
mtlsu.at	0.861	12145.502
mtlsu.cg	0.475	11719.324
mtlsu.gt	1.037	10058.615
mtlsu.frequencies1	0.259	13149.532
mtlsu.frequencies2	0.232	12865.207
mtlsu.frequencies3	0.284	12123.031
mtlsu.frequencies4	0.225	13316.919
mtlsu.alpha	0.291	22709.683
mtlsu.pInv	0.465	24381.669
mtssu.ac	0.798	10155.545
mtssu.ag	0.438	7992.721
mtssu.at	0.629	10282.256
mtssu.cg	0.291	12815.774
mtssu.gt	0.883	10026.358
mtssu.frequencies1	0.252	12670.605
mtssu.frequencies2	0.232	12747.647
mtssu.frequencies3	0.308	12954.708
mtssu.frequencies4	0.208	12316.354
mtssu.alpha	0.292	15552.801
mtssu.pInv	0.42	16028.209
ndhf.ac	0.366	8699.309
ndhf.ag	1.225	4457.707
ndhf.at	0.09255	10076.403

ndhf.cg	0.433	10813.954
ndhf.gt	0.27	8539.855
ndhf.frequencies1	0.301	5375.134
ndhf.frequencies2	0.152	6571.797
ndhf.frequencies3	0.143	6129.975
ndhf.frequencies4	0.404	5024.068
ndhf.alpha	0.831	6899.146
ndhf.pInv	0.331	6695.725
rbcl.ac	0.363	9777.348
rbcl.ag	0.794	4553.653
rbcl.at	0.124	3663.926
rbcl.cg	0.269	8867.217
rbcl.gt	0.21	7112.924
rbcl.frequencies1	0.279	4399.109
rbcl.frequencies2	0.182	6132.125
rbcl.frequencies3	0.236	4009.724
rbcl.frequencies4	0.302	7018.436
rbcl.alpha	0.61	16760.607
rbcl.pInv	0.442	16007.945
trnlfspr.ac	0.456	4581.736
trnlfspr.ag	0.844	3137.789
trnlfspr.at	0.195	5031.204
trnlfspr.cg	0.363	7742.739
trnlfspr.gt	0.368	6796.917
trnlfspr.frequencies1	0.33	3516.917
trnlfspr.frequencies2	0.162	4897.295
trnlfspr.frequencies3	0.169	5788.42
trnlfspr.frequencies4	0.34	3713.054
trnlfspr.alpha	0.878	19541.008
trnli.ac	0.374	8954.925
trnli.ag	0.773	5392.239
trnli.at	0.14	10609.054
trnli.cg	0.307	13053.479
trnli.gt	0.466	8589.553
trnli.frequencies1	0.404	6804.078
trnli.frequencies2	0.146	7246.069
trnli.frequencies3	0.177	8221.773
trnli.frequencies4	0.273	6661.827
trnli.alpha	1.908	19568.141
trnli.pInv	0.206	22083.774
ucld.mean	0.0005007	131.977
ucld.stdev	0.824	158.196
meanRate	0.0004598	153.86
coefficientOfVariation	1.041	215.152
covariance	0.218	358.367
18s.treeLikelihood	-9411.873	7155.312
26s.treeLikelihood	-24135.69	9562.951
atp1.treeLikelihood	-6078.44	5318.38
atpb.treeLikelihood	-11797.583	9972.719
matk.treeLikelihood	-19839.109	2458.191

matr.treeLikelihood	-9752.734	6689.925
mtlsu.treeLikelihood	-11106.24	10505.481
mtssu.treeLikelihood	-7532.597	7832.335
ndhf.treeLikelihood	-32007.245	8672.527
rbcl.treeLikelihood	-18833.415	671.517
trnlfspr.treeLikelihood	-12388.899	1420.693
trnli.treeLikelihood	-8589.954	1162.465
speciation	-1026.589	136.026

Angio 150

**Run**	**Burnin in million generations**	**Frequency used to summarize results from tree files in number of generations**
1	10	15,000
2	10	
3	10	
4	10	

**Statistic of the combined four runs**	**Mean**	**ESS**
posterior	-174300	682.997
prior	-2800.756	152.012
likelihood	-171500	4823.455
treeModel.rootHeight	149.283	3306.359
tmrca(All)	149.283	3306.359
tmrca(Caly_Chimo)	89.228	570.506
tmrca(Canellales)	127.072	877.622
tmrca(core_Laurales)	111.173	1162.431
tmrca(Laurales)	119.905	264.353
tmrca(Saur_Gymno)	50.719	708.064
tmrca(eudicots)	106.408	168.977
tmrca(root)	148.489	1639.582
tmrca(Magnoliinae)	115.803	514.17
birthDeath.meanGrowthRate	0.02665	853.242
birthDeath.relativeDeathRate	0.362	8530.158
birthDeath.sampleProbability	0.556	1499.282
18s.ac	0.186	16942.54
18s.ag	0.346	10136.766
18s.at	0.213	17613.79
18s.cg	0.104	17848.406
18s.gt	0.141	17797.857
18s.frequencies1	0.242	10939.918
18s.frequencies2	0.232	12178.382
18s.frequencies3	0.267	12599.942
18s.frequencies4	0.26	11096.299
18s.alpha	0.325	13138.243
18s.pInv	0.484	12983.526
26s.ac	0.09262	12895.213
26s.ag	0.385	7146.006

26s.at	0.187	17120.31
26s.cg	0.05946	14689.813
26s.gt	0.127	18538.427
26s.frequencies1	0.263	8075.128
26s.frequencies2	0.236	9107.128
26s.frequencies3	0.309	10810.091
26s.frequencies4	0.192	7293.158
26s.alpha	0.602	6922.691
26s.pInv	0.215	6769.187
atp1.ac	0.758	14327.888
atp1.ag	0.813	9742.76
atp1.at	0.776	13222.555
atp1.cg	0.3	15978.948
atp1.gt	0.388	13503.537
atp1.frequencies1	0.28	10989.012
atp1.frequencies2	0.206	12289.039
atp1.frequencies3	0.244	13609.507
atp1.frequencies4	0.27	12154.304
atp1.alpha	0.293	10060.897
atp1.pInv	0.415	10108.371
atpb.ac	0.233	16443.259
atpb.ag	0.796	10239.165
atpb.at	0.07541	20142.706
atpb.cg	0.147	21796.634
atpb.gt	0.122	18584.199
atpb.frequencies1	0.293	10623.655
atpb.frequencies2	0.203	10845.008
atpb.frequencies3	0.235	10447.345
atpb.frequencies4	0.27	11732.629
atpb.alpha	0.505	5364.669
atpb.pInv	0.31	5399.127
matk.ac	0.455	5726.134
matk.ag	1.051	4236.756
matk.at	0.06822	13689.313
matk.cg	0.307	8692.895
matk.gt	0.309	8048.397
matk.frequencies1	0.29	5024.534
matk.frequencies2	0.151	8028.852
matk.frequencies3	0.153	5636.997
matk.frequencies4	0.406	4202.062
matk.alpha	1.149	10319.859
matk.pInv	0.136	10158.54
matr.ac	0.959	12012.41
matr.ag	0.629	8951.223
matr.at	0.724	14313.73
matr.cg	0.249	13068.392
matr.gt	0.839	11249.753
matr.frequencies1	0.263	11606.123
matr.frequencies2	0.266	12206.509
matr.frequencies3	0.254	12525.417

matr.frequencies4	0.217	11434.521
matr.alpha	0.354	12645.932
matr.pInv	0.09559	11956.936
mtlsu.ac	1.248	11450.194
mtlsu.ag	0.625	8320.103
mtlsu.at	0.862	10635.091
mtlsu.cg	0.476	11536.703
mtlsu.gt	1.04	9927.946
mtlsu.frequencies1	0.259	12772.806
mtlsu.frequencies2	0.232	13086.524
mtlsu.frequencies3	0.284	13236.828
mtlsu.frequencies4	0.225	13254.639
mtlsu.alpha	0.291	23116.323
mtlsu.pInv	0.465	24179.817
mtssu.ac	0.798	8280.527
mtssu.ag	0.437	6998.592
mtssu.at	0.629	9118.586
mtssu.cg	0.291	11863.698
mtssu.gt	0.882	9432.42
mtssu.frequencies1	0.252	13490.278
mtssu.frequencies2	0.232	12917.804
mtssu.frequencies3	0.308	12589.284
mtssu.frequencies4	0.207	13137.869
mtssu.alpha	0.292	16078.665
mtssu.pInv	0.42	16118.395
ndhf.ac	0.367	8512.226
ndhf.ag	1.228	4662.361
ndhf.at	0.09267	10821.754
ndhf.cg	0.434	10866.871
ndhf.gt	0.27	8800.669
ndhf.frequencies1	0.301	5240.343
ndhf.frequencies2	0.152	6713.041
ndhf.frequencies3	0.143	6250.713
ndhf.frequencies4	0.404	4873.839
ndhf.alpha	0.834	7590.262
ndhf.pInv	0.332	7409.446
rbcl.ac	0.363	7415.9
rbcl.ag	0.797	4211.835
rbcl.at	0.124	7667.605
rbcl.cg	0.27	9730.464
rbcl.gt	0.211	6801.567
rbcl.frequencies1	0.28	5764.663
rbcl.frequencies2	0.182	5772.387
rbcl.frequencies3	0.236	6218.225
rbcl.frequencies4	0.302	6263.654
rbcl.alpha	0.61	16060.506
rbcl.pInv	0.442	15909.036
trnlfspr.ac	0.459	4136.467
trnlfspr.ag	0.847	2887.253
trnlfspr.at	0.195	4915.198

trnlfspr.cg	0.364	6862.053
trnlfspr.gt	0.368	6314.997
trnlfspr.frequencies1	0.329	3322.526
trnlfspr.frequencies2	0.162	4990.031
trnlfspr.frequencies3	0.169	5667.095
trnlfspr.frequencies4	0.341	3471.615
trnlfspr.alpha	0.877	20733.962
trnli.ac	0.375	8288.722
trnli.ag	0.773	5175.462
trnli.at	0.14	10708.764
trnli.cg	0.307	11559.418
trnli.gt	0.466	8601.828
trnli.frequencies1	0.404	6173.652
trnli.frequencies2	0.146	7532.593
trnli.frequencies3	0.177	7626.883
trnli.frequencies4	0.273	6138.13
trnli.alpha	1.914	19251.415
trnli.pInv	0.207	21267.931
ucld.mean	0.0004697	138.037
ucld.stdev	0.748	247.498
meanRate	0.0004506	165.706
coefficientOfVariation	0.876	238.658
covariance	0.165	440.809
18s.treeLikelihood	-9411.912	3148.83
26s.treeLikelihood	-24136.239	7144.017
atp1.treeLikelihood	-6078.563	7602.37
atpb.treeLikelihood	-11797.425	8658.379
matk.treeLikelihood	-19839.34	2370.236
matr.treeLikelihood	-9752.753	4391.17
mtlsu.treeLikelihood	-11106.334	12501.091
mtssu.treeLikelihood	-7532.641	10010.464
ndhf.treeLikelihood	-32007.166	9197.638
rbcl.treeLikelihood	-18831.815	450.389
trnlfspr.treeLikelihood	-12388.332	1240.787
trnli.treeLikelihood	-8589.546	1448.395
speciation	-1031.223	146.006

Angio 170

**Run**	**Burnin in million generations**	**Frequency used to summarize results from tree files in number of generations**
1	10	15,000
2	10	
3	15	
4	15	

**Statistic of the combined four runs**	**Mean**	**ESS**
posterior	-174300	724.88

prior	-2810.944	174.565
likelihood	-171500	5852.575
treeModel.rootHeight	168.321	1878.826
tmrca(All)	168.321	1878.826
tmrca(Caly_Chimo)	89.142	786.058
tmrca(Canellales)	128.84	505.002
tmrca(core_Laurales)	112.509	1180.179
tmrca(Laurales)	123.955	345.616
tmrca(Saur_Gymno)	51.539	568.131
tmrca(eudicots)	110.839	195.43
tmrca(root)	166.649	964.665
tmrca(Magnoliinae)	117.695	422.452
birthDeath.meanGrowthRate	0.02504	980.499
birthDeath.relativeDeathRate	0.367	9693.7
birthDeath.sampleProbability	0.569	1749.104
18s.ac	0.186	16339.116
18s.ag	0.346	9916.702
18s.at	0.213	17210.68
18s.cg	0.105	16214.675
18s.gt	0.141	16444.484
18s.frequencies1	0.242	10965.085
18s.frequencies2	0.232	11345.615
18s.frequencies3	0.266	11544.862
18s.frequencies4	0.26	9860.608
18s.alpha	0.324	12960.797
18s.pInv	0.483	12729.293
26s.ac	0.09242	11264.249
26s.ag	0.385	6712.361
26s.at	0.186	15815.169
26s.cg	0.05936	12956.079
26s.gt	0.127	19097.949
26s.frequencies1	0.263	6691.715
26s.frequencies2	0.236	9494.939
26s.frequencies3	0.309	9912.96
26s.frequencies4	0.192	6605.991
26s.alpha	0.603	7129.751
26s.pInv	0.216	6967.256
atp1.ac	0.76	14773.329
atp1.ag	0.817	9406.458
atp1.at	0.778	12952.856
atp1.cg	0.301	15109.508
atp1.gt	0.389	12668.808
atp1.frequencies1	0.28	10729.37
atp1.frequencies2	0.206	11867.609
atp1.frequencies3	0.244	12605.621
atp1.frequencies4	0.27	12173.674
atp1.alpha	0.294	9799.75
atp1.pInv	0.417	9778.589
atpb.ac	0.233	16019.81
atpb.ag	0.796	10422.588

atpb.at	0.07542	20811.196
atpb.cg	0.147	20691.305
atpb.gt	0.122	17872.524
atpb.frequencies1	0.292	11486.716
atpb.frequencies2	0.203	11616.255
atpb.frequencies3	0.235	11756.257
atpb.frequencies4	0.27	11153.23
atpb.alpha	0.504	5231.494
atpb.pInv	0.309	5262.427
matk.ac	0.456	6000.097
matk.ag	1.053	4176.667
matk.at	0.06834	12291.522
matk.cg	0.307	8219.317
matk.gt	0.309	8278.8
matk.frequencies1	0.29	4649.116
matk.frequencies2	0.151	7307.245
matk.frequencies3	0.153	5030.989
matk.frequencies4	0.406	4213.758
matk.alpha	1.15	10090.337
matk.pInv	0.136	9974.556
matr.ac	0.958	10935.233
matr.ag	0.627	8442.96
matr.at	0.722	12597.033
matr.cg	0.248	12925.977
matr.gt	0.837	10945.231
matr.frequencies1	0.263	10841.104
matr.frequencies2	0.266	11603.662
matr.frequencies3	0.254	11927.649
matr.frequencies4	0.217	10497.94
matr.alpha	0.354	10651.629
matr.pInv	0.09543	10955.546
mtlsu.ac	1.249	11889.897
mtlsu.ag	0.626	8695.859
mtlsu.at	0.863	11203.223
mtlsu.cg	0.476	11856.649
mtlsu.gt	1.042	9915.562
mtlsu.frequencies1	0.259	12554.131
mtlsu.frequencies2	0.232	12105.016
mtlsu.frequencies3	0.284	10547.357
mtlsu.frequencies4	0.225	12447.716
mtlsu.alpha	0.291	21143.691
mtlsu.pInv	0.465	21298.303
mtssu.ac	0.8	10551.131
mtssu.ag	0.438	8496.86
mtssu.at	0.629	11352.822
mtssu.cg	0.291	12925.67
mtssu.gt	0.881	11301.292
mtssu.frequencies1	0.252	12115.264
mtssu.frequencies2	0.233	12736.512
mtssu.frequencies3	0.308	13278.014

mtssu.frequencies4	0.208	12709.773
mtssu.alpha	0.293	13787.216
mtssu.pInv	0.421	14394.395
ndhf.ac	0.366	8535.744
ndhf.ag	1.225	4413.946
ndhf.at	0.09268	9574.651
ndhf.cg	0.433	11370.655
ndhf.gt	0.27	8181.531
ndhf.frequencies1	0.301	5024.786
ndhf.frequencies2	0.152	6649.782
ndhf.frequencies3	0.143	6160.654
ndhf.frequencies4	0.404	5184.459
ndhf.alpha	0.835	7055.637
ndhf.pInv	0.332	6902.576
rbcl.ac	0.365	8507.889
rbcl.ag	0.798	4439.611
rbcl.at	0.123	8310.201
rbcl.cg	0.271	11199.704
rbcl.gt	0.211	7595.08
rbcl.frequencies1	0.28	5960.237
rbcl.frequencies2	0.182	5103.988
rbcl.frequencies3	0.236	7080.863
rbcl.frequencies4	0.303	6758.719
rbcl.alpha	0.611	17993.693
rbcl.pInv	0.443	17135.374
trnlfspr.ac	0.459	4109.11
trnlfspr.ag	0.847	2718.81
trnlfspr.at	0.195	3442.304
trnlfspr.cg	0.364	7095.45
trnlfspr.gt	0.368	5607.633
trnlfspr.frequencies1	0.329	3310.008
trnlfspr.frequencies2	0.162	3919.011
trnlfspr.frequencies3	0.169	5491.239
trnlfspr.frequencies4	0.34	3145.69
trnlfspr.alpha	0.877	18871.435
trnli.ac	0.374	8861.113
trnli.ag	0.771	5457.21
trnli.at	0.14	11275.275
trnli.cg	0.307	11261.381
trnli.gt	0.465	8336.783
trnli.frequencies1	0.404	6797.377
trnli.frequencies2	0.146	8077.475
trnli.frequencies3	0.178	6855.91
trnli.frequencies4	0.272	6464.388
trnli.alpha	1.908	19711.417
trnli.pInv	0.206	21426.191
ucld.mean	0.0004312	170.511
ucld.stdev	0.695	217.171
meanRate	0.00043	190.445
coefficientOfVariation	0.786	198.818

covariance	0.116	404.965
18s.treeLikelihood	-9411.963	8621.072
26s.treeLikelihood	-24137.081	8372.297
atp1.treeLikelihood	-6078.371	4327.734
atpb.treeLikelihood	-11797.291	6580.839
matk.treeLikelihood	-19837.549	2497.778
matr.treeLikelihood	-9753.167	9017.865
mtlsu.treeLikelihood	-11106.295	10570.491
mtssu.treeLikelihood	-7532.67	3584.558
ndhf.treeLikelihood	-32007.744	9360.257
rbcl.treeLikelihood	-18835.464	585.929
trnlfspr.treeLikelihood	-12387.214	1171.63
trnli.treeLikelihood	-8588.15	1527.602
speciation	-1041.449	168.44

Angio 200

**Run**	**Burnin in million generations**	**Frequency used to summarize results from tree files in number of generations**
1	10	15,000
2	10	
3	10	
4	10	

**Statistic of the combined four runs**	**Mean**	**ESS**
posterior	-174300	1072.256
prior	-2827.13	244.696
likelihood	-171500	10662.375
treeModel.rootHeight	196.25	1263.293
tmrca(All)	196.25	1263.293
tmrca(Caly_Chimo)	89.639	851.913
tmrca(Canellales)	132.426	389.164
tmrca(core_Laurales)	114.755	723.94
tmrca(Laurales)	131.116	351.532
tmrca(Saur_Gymno)	52.248	811.569
tmrca(eudicots)	116.137	308.126
tmrca(root)	192.834	619.204
tmrca(Magnoliinae)	122.457	236.257
birthDeath.meanGrowthRate	0.02298	1164.317
birthDeath.relativeDeathRate	0.37	10959.131
birthDeath.sampleProbability	0.58	2326.192
18s.ac	0.187	17134.978
18s.ag	0.348	11315.475
18s.at	0.213	18103.518
18s.cg	0.105	18196.765
18s.gt	0.141	16971.027
18s.frequencies1	0.242	11368.171
18s.frequencies2	0.232	12286.947

18s.frequencies3	0.266	12400.481
18s.frequencies4	0.26	11116.226
18s.alpha	0.324	13073.953
18s.pInv	0.484	12834.211
26s.ac	0.09261	12071.025
26s.ag	0.385	7363.637
26s.at	0.187	17627.321
26s.cg	0.05937	14343.082
26s.gt	0.127	21621.147
26s.frequencies1	0.263	7894.137
26s.frequencies2	0.236	10976.429
26s.frequencies3	0.309	11106.572
26s.frequencies4	0.192	7374.31
26s.alpha	0.601	6125.38
26s.pInv	0.215	5873.809
atp1.ac	0.76	15678.463
atp1.ag	0.816	9954.818
atp1.at	0.777	13500.64
atp1.cg	0.301	16814.159
atp1.gt	0.389	13847.352
atp1.frequencies1	0.28	11338.293
atp1.frequencies2	0.206	12302.184
atp1.frequencies3	0.243	13919.953
atp1.frequencies4	0.27	12722.663
atp1.alpha	0.294	8864.896
atp1.pInv	0.416	9529.207
atpb.ac	0.233	16226.384
atpb.ag	0.795	10028.998
atpb.at	0.07539	21544.148
atpb.cg	0.147	20421.054
atpb.gt	0.122	18658.263
atpb.frequencies1	0.293	11078.262
atpb.frequencies2	0.203	11912.395
atpb.frequencies3	0.235	12052.689
atpb.frequencies4	0.27	11668.311
atpb.alpha	0.503	5675.101
atpb.pInv	0.309	5691.378
matk.ac	0.456	5544.303
matk.ag	1.052	3949.181
matk.at	0.06832	10324.045
matk.cg	0.307	8859.259
matk.gt	0.309	8197.883
matk.frequencies1	0.29	4400.147
matk.frequencies2	0.151	7725.371
matk.frequencies3	0.153	6006.871
matk.frequencies4	0.406	4118.561
matk.alpha	1.148	10987.022
matk.pInv	0.136	10682.94
matr.ac	0.958	11443.278
matr.ag	0.627	8682.954

matr.at	0.723	13847.739
matr.cg	0.248	13184.272
matr.gt	0.838	11617.031
matr.frequencies1	0.263	11489.369
matr.frequencies2	0.266	12827.034
matr.frequencies3	0.254	12425.73
matr.frequencies4	0.217	10928.844
matr.alpha	0.354	11095.054
matr.pInv	0.09558	10565.062
mtlsu.ac	1.244	11829.496
mtlsu.ag	0.622	8850.613
mtlsu.at	0.861	12157.436
mtlsu.cg	0.474	11808.768
mtlsu.gt	1.037	10295.182
mtlsu.frequencies1	0.259	12482.191
mtlsu.frequencies2	0.232	13181.878
mtlsu.frequencies3	0.284	12158.581
mtlsu.frequencies4	0.225	11875.223
mtlsu.alpha	0.291	21315.46
mtlsu.pInv	0.465	22081.134
mtssu.ac	0.8	10053.385
mtssu.ag	0.439	7700.097
mtssu.at	0.63	10468.876
mtssu.cg	0.291	12072.275
mtssu.gt	0.885	10189.333
mtssu.frequencies1	0.252	11475.067
mtssu.frequencies2	0.233	12085.22
mtssu.frequencies3	0.308	11164.33
mtssu.frequencies4	0.208	11085.854
mtssu.alpha	0.292	15290.854
mtssu.pInv	0.421	15005.494
ndhf.ac	0.366	10026.956
ndhf.ag	1.224	4718.419
ndhf.at	0.09253	11200.09
ndhf.cg	0.434	11503.177
ndhf.gt	0.27	8815.829
ndhf.frequencies1	0.302	5996.79
ndhf.frequencies2	0.152	6927.982
ndhf.frequencies3	0.143	6207.812
ndhf.frequencies4	0.404	5813.32
ndhf.alpha	0.833	6691.614
ndhf.pInv	0.331	6229.654
rbcl.ac	0.364	8674.588
rbcl.ag	0.802	4523.041
rbcl.at	0.124	9323.777
rbcl.cg	0.271	10412.757
rbcl.gt	0.213	7356.128
rbcl.frequencies1	0.28	6610.219
rbcl.frequencies2	0.183	6288.084
rbcl.frequencies3	0.235	6428.955

rbcl.frequencies4	0.302	6692.088
rbcl.alpha	0.612	17277.89
rbcl.pInv	0.442	16939.11
trnlfspr.ac	0.457	4254.12
trnlfspr.ag	0.842	2033.363
trnlfspr.at	0.195	3264.406
trnlfspr.cg	0.363	6416.873
trnlfspr.gt	0.368	5053.178
trnlfspr.frequencies1	0.33	2544.266
trnlfspr.frequencies2	0.162	3916.033
trnlfspr.frequencies3	0.169	4808.771
trnlfspr.frequencies4	0.34	3512.899
trnlfspr.alpha	0.876	21662.105
trnli.ac	0.374	8927.874
trnli.ag	0.771	5553.63
trnli.at	0.14	11221.426
trnli.cg	0.307	11960.598
trnli.gt	0.465	8857.653
trnli.frequencies1	0.405	6559.955
trnli.frequencies2	0.146	7636.452
trnli.frequencies3	0.178	7140.339
trnli.frequencies4	0.272	6204.307
trnli.alpha	1.91	19096.588
trnli.pInv	0.207	19304.541
ucld.mean	0.0004007	198.642
ucld.stdev	0.677	231.355
meanRate	0.0003995	262.022
coefficientOfVariation	0.74	266.886
covariance	0.09617	393.231
18s.treeLikelihood	-9411.327	7954.215
26s.treeLikelihood	-24137.495	9824.141
atp1.treeLikelihood	-6077.993	6580.656
atpb.treeLikelihood	-11797.017	10871.194
matk.treeLikelihood	-19838.952	2728.554
matr.treeLikelihood	-9753.026	7063.446
mtlsu.treeLikelihood	-11106.292	7546.342
mtssu.treeLikelihood	-7532.69	10590.07
ndhf.treeLikelihood	-32008.545	9489.979
rbcl.treeLikelihood	-18831.121	642.502
trnlfspr.treeLikelihood	-12388.804	1275.412
trnli.treeLikelihood	-8588.839	1723.789
speciation	-1057.529	236.882
