# Supplementary material for: Five major shifts of diversification through the long evolutionary history of Magnoliidae (angiosperms)
Source: BMC Evol Biol. 2015 Mar 18;15:49. doi: 10.1186/s12862-015-0320-6 (PMC4377182; doi:10.1186/s12862-015-0320-6)

Additional file 2. Illustration of the compartments for the diversification analyses. Tree modified from the maximum clade credibility tree of the BEAST angio-140 analysis (branch times contracted in some clades for graphical purposes). Numbers at nodes are posterior probability values, which are compatible with all other BEAST analyses. Green terminals are the compartments used for the diversification analysis. Braces highlight clades in our chronograms collapsed as terminals. We used the abbreviation 'incl.' to indicate where the species richness of unsampled genera has been counted, and 'excl.' where it was ignored.

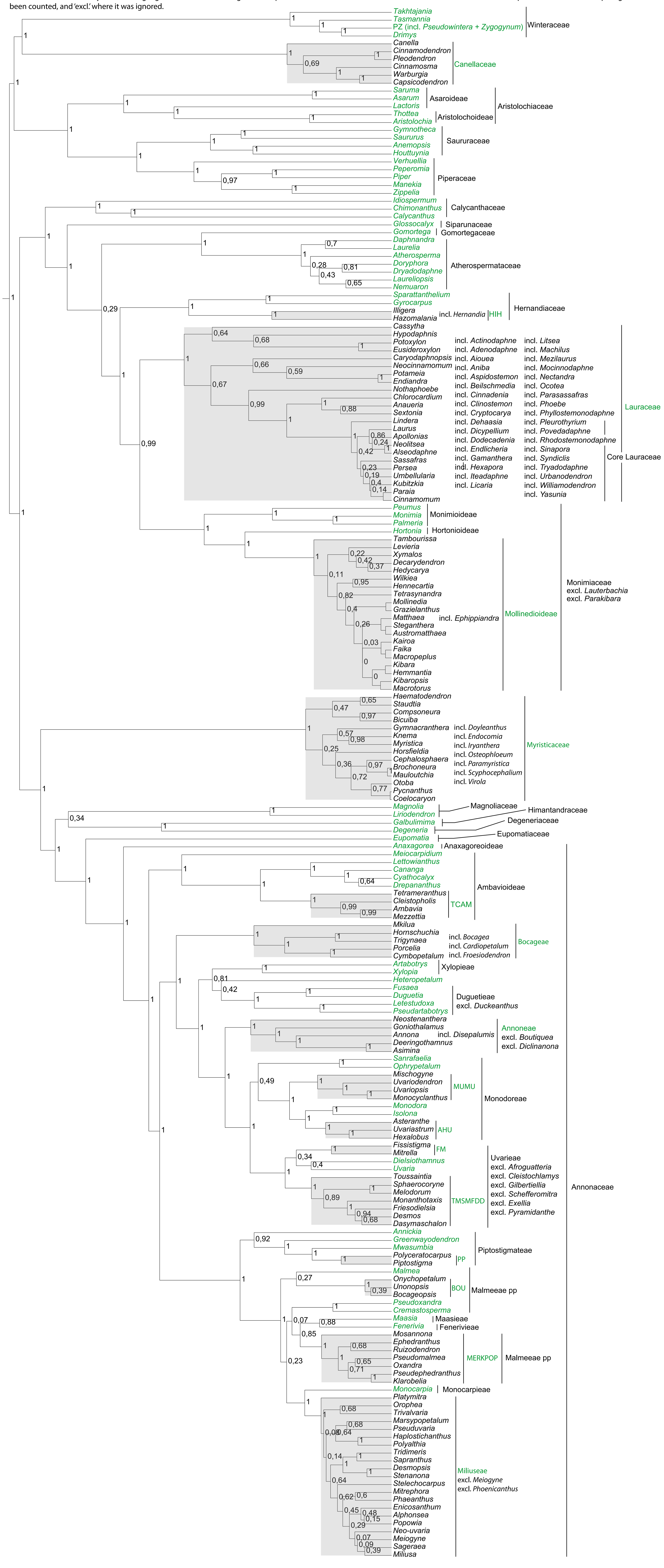

Supplement: Additional file 2: — Illustration of the compartments for the diversification analyses. Tree modified from the maximum clade credibility tree of the BEAST angio-140 analysis (branch times contracted in some clades for graphical purposes). Numbers at nodes are posterior probability values, which are compatible with all other BEAST analyses. Green terminals are the compartments used for the diversification analysis. Braces highlight clades in our chronograms collapsed as terminals. We used the abbreviation ‘incl.’ to indicate where the species richness of unsampled genera has been counted, and ‘excl.’ where it was ignored. [file 12862_2015_320_MOESM2_ESM.pdf]
