# Supplementary material for: Five major shifts of diversification through the long evolutionary history of Magnoliidae (angiosperms)
Source: BMC Evol Biol. 2015 Mar 18;15:49. doi: 10.1186/s12862-015-0320-6 (PMC4377182; doi:10.1186/s12862-015-0320-6)
Supplement: Additional file 4: — Relative extinction rates. Same figure as Figure 2, but illustrating variation in relative extinction rates. Results of the MEDUSA analyses obtained using 1000 chronograms randomly sampled from the BEAST posterior: A, angio-140, B, angio-200. The topologies are the maximum clade credibility trees of the 1000 trees used. Names of leaves refer to terminal compartments defined to conduct this analysis. Branch colors illustrate the mean relative rate of extinction (epsilon). Red dots denote significant shifts in net diversification rate (r), their size being proportional to their frequency among the 1000 trees tested. Abbreviation: Myr, million years. [file 12862_2015_320_MOESM4_ESM.pdf]

Additional file 4. Relative extinction rates. Same figure as Fig. 3, but illustrating variation in relative extinction rates. Results of the MEDUSA analyses obtained using 1000 chronograms randomly sampled from the BEAST posterior: A, angio-140, B, angio-200. The topologies are the maximum clade credibility trees of the 1000 trees used. Names of leaves refer to terminal compartments defined to conduct this analysis. Branch colors illustrate the mean relative rate of extinction (epsilon). Red dots denote significant shifts in net diversification rate (r), their size being proportional to their frequency among the 1000 trees tested. Abbreviation: Myr, million years.

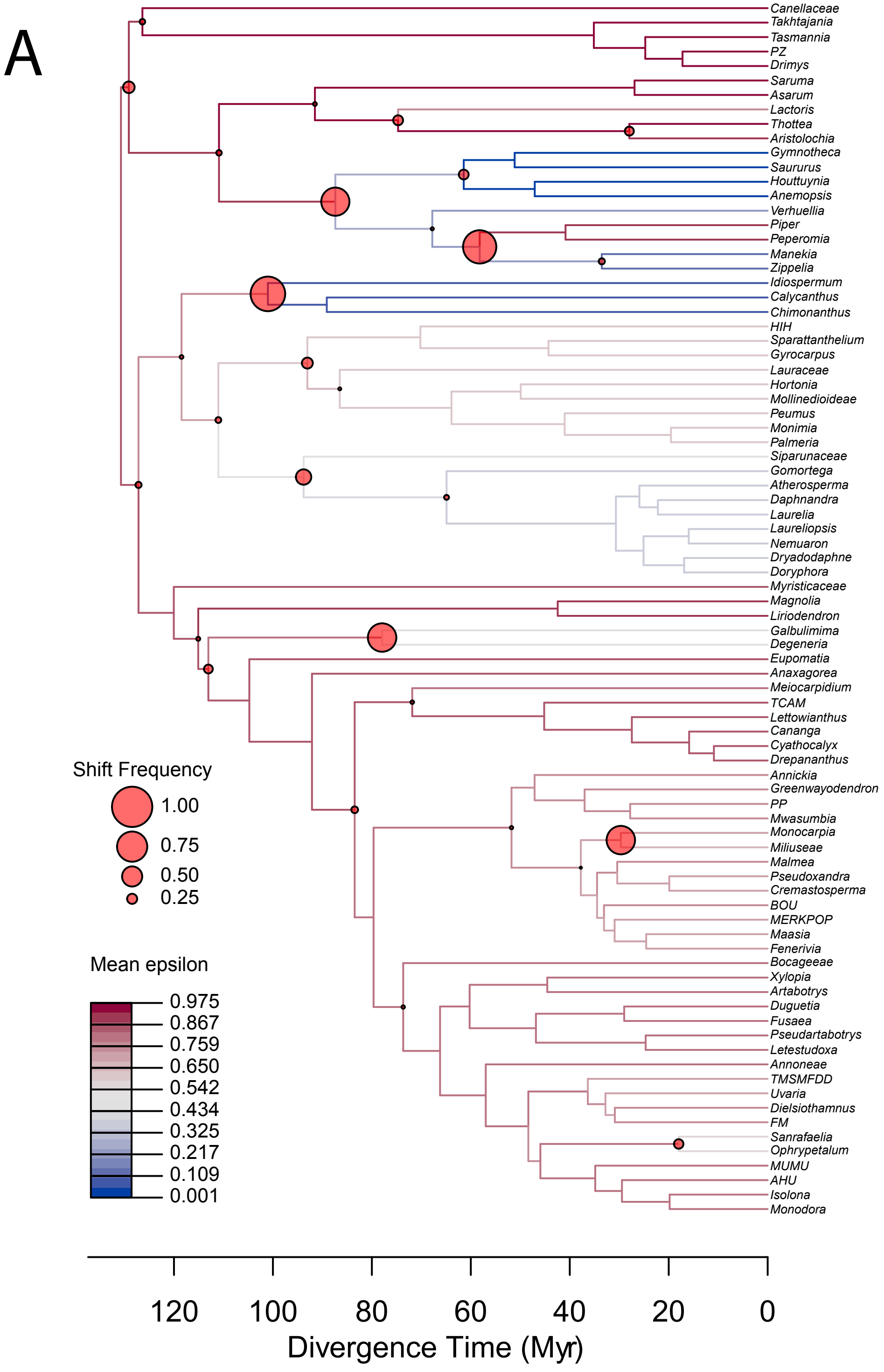

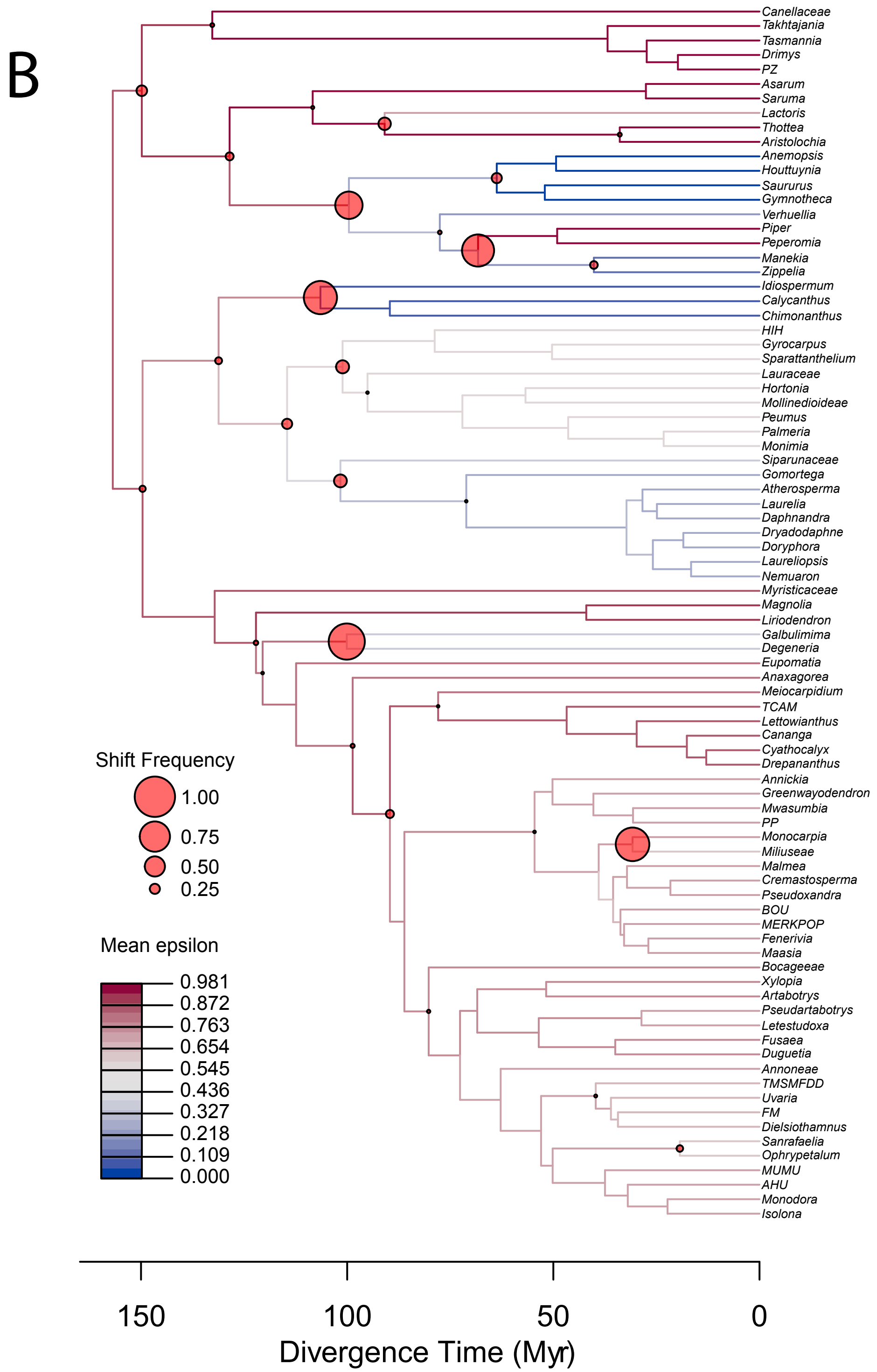
B
Canellaceae
Takhtajania
Tasmannia
Drimys
PZ
Asarum
Saruma
Lactoris
Thottea
Aristolochia
Anemopsis
Houttuynia
Saururus
Gymnotheca
Verhuellia
Piper
Peperomia
Manekia
Zippelia
Idiospermum
Calycanthus
Chimonanthus
HIH
Gyrocarpus
Sparattanthelium
Lauraceae
Hortonia
Mollinedioideae
Peumus
Palmeria
Monimia
Siparunaceae
Gomortega
Atherosperma
Laurelia
Daphnandra
Dryadodaphne
Doryphora
Laureliopsis
Nemuaron
Myristicaceae
Magnolia
Liriodendron
Galbulimima
Degeneria
Eupomatia
Anaxagorea
Meiocarpidium
TCAM
Lettowianthus
Cananga
Cyathocalyx
Drepananthus
Annickia
Greenwayodendron
Mwasumbia
PP
Monocarpia
Miliuseae
Malmea
Cremastosperma
Pseudoxandra
BOU
MERKPOP
Fenerivia
Maasia
Bocageeae
Xylopia
Artabotrys
Pseudartabotrys
Letestudoxa
Fusaea
Duguetia
Annoneae
TMSMFDD
Uvaria
FM
Dielsiothamnus
Sanrafaelia
Ophrypetalum
MUMU
AHU
Monodora
Isolona
Shift Frequency
1.00
0.75
0.50
0.25
Mean epsilon
0.981
0.872
0.763
0.654
0.545
0.436
0.327
0.218
0.109
0.000
150
100
50
0
Divergence Time (Myr)
