# Supplementary material for: Five major shifts of diversification through the long evolutionary history of Magnoliidae (angiosperms)
Source: BMC Evol Biol. 2015 Mar 18;15:49. doi: 10.1186/s12862-015-0320-6 (PMC4377182; doi:10.1186/s12862-015-0320-6)
Supplement: Additional file 5: — Definition of compartments for diversification analyses. This appendix provides tables justifying the monophyly and the number of species within compartments. In addition, a text provides all justifications for the definition of supra-generic compartments, for assignment of species richness of genera not sampled in our chronograms, and for occasionally ignoring such missing genera. [file 12862_2015_320_MOESM5_ESM.pdf]

Additional file 5. Definition of compartments for the diversification rate analysis.

This appendix provides tables justifying the monophyly and the number of species within compartments. In addition, a text provides all justifications for the definition of supra-generic compartments, for assignment of species richness of genera not sampled in our chronograms, and for occasionally ignoring such missing genera. Additional file 2 illustrates the present appendix. The posterior probabilities (PP) presented in this figure are similar in all other BEAST analyses. Abbreviations: nb., number; sp., species; ref., reference.

Annonaceae

**Compartment**	**Compartment content**	**Nb. of sp. in genus**	**Ref. for sp. content**	**Nb. of sp. in compartment**	**Ref. for the monophyly of the compartment**
AHU	*Asteranthe*	2	[1]	12	[2] / [1] / [3]
	Hexalobus	5	[1]		
	Uvariastrum	5	[4]		
Anaxagorea	-	30	[1]	30	[5]
Annickia	-	8	[1]	8	[6] / [7]
Annoneae	*Annona*	162	[1]	321	[8] / [1] / [3]
	Anonidium	4	[1]		
	Asimina (including *Deeringothamus*)	7	[1]		
	Disepalum	9	[1]		
	Goniothalamus	134	[1]		
	Neostenanthera	5	[9]		
Artabotrys	-	102	[1]	102	[10]
Bocageeae	*Bocagea*	2	[1]	65	[1] / [3]
	Cardiopetalum	3	[1]		
	Cymbopetalum	27	[1]		

	Froesiodendron	3	[1]		
	Hornschuchia	10	[1]		
	Mkilua	1	[1]		
	Porcelia	7	[1]		
	Trigynaea	12	[1]		
BOU	*Bocageopsis*	4	[1]	54	[8] / [1] / [11] / [3]
	Onychopetalum	2	[1]		
	Unonopsis	48	[1]		
Cananga	-	2	[1]	2	[12]
Cremastosperma	-	29	[1]	29	[6]
Cyathocalyx	-	7	[1]	7	[12]
Dielsiothamnus	-	1	[1]	1	-
Drepananthus	-	26	[1]	26	[12]
Duguetia	-	93	[1]	93	[6]
Fenerivia	-	10	[1]	10	[13]
FM	*Fissistigma*	48	[1]	56	[1] / [3]
	Mitrella	8	[1]		
Fusaea	-	2	[1]	2	[1]
Greenwayodendron	-	2	[1]	2	[14]
Guatteria	-	210	[1]	210	[15]
Isolona	-	20	[1]	20	[16]
Letestudoxa	-	3	[1]	3	[8]
Lettowianthus	-	1	[1]	1	-
Maasia	-	6	[1]	6	[13]
Malmea	-	6	[1]	6	[14]
Meiocarpidium	-	1	[1]	1	-
MERKPOP	*Ephedranthus*	6	[1]	66	[7] / [17] / [11] / [3]
	Klarobelia	12	[1]		
	Mosannona	14	[1]		
	Oxandra	28	[1]		
	Pseudephedranthus	1	[1]		
	Pseudomalmea	4	[1]		
	Ruizodendron	1	[1]		
Miliuseae	*Alphonsea*	25	[1]	510	[1] / [11] / [3]
	Desmopsis	14	[1]		
	Enicosanthum	18	[1]		
	Haplostichanthus	11	[1]		

	Marsypopetalum	6	[1]		
	Meiogyne (including *Fitzalania*)	17	[18]		
	Miliusa	50	[1]		
	Mitrephora	47	[1]		
	Neo-uvaria	5	[1]		
	Orophea	50	[1]		
	Phaeanthus	9	[1]		
	Platymitra	2	[1]		
	Polyalthia	135	[1]		
	Popowia	26	[1]		
	Pseuduvaria	57	[1]		
	Sageraea	9	[1]		
	Sapranthus	6	[1]		
	Stelechocarpus	3	[1]		
	Stenanona	14	[1]		
	Tridimeris	1	[1]		
	Trivalvaria	4	[1]		
	Woodiellantha	1	[1]		
Monocarpia	-	1	[1]	1	-
Monodora	-	16	[1]	16	[16]
MUMU	*Mischogyne*	2	[1]	34	[2] / [1] / [3]
	Monocyclanthus	1	[1]		
	Uvariodendron	15	[1]		
	Uvariopsis	16	[1]		
Mwasumbia	-	1	[1]	1	-
Ophrypetalum	-	1	[1]	1	-
PP	*Piptostigma*	14	[1]	22	[7]
	Polyceratocarpus	8	[1]		
Pseudartabotrys	-	1	[1]	1	-
Pseudoxandra	-	23	[1]	23	[14]
Sanrafaelia	-	1	[1]	1	-
TCAM	*Ambavia*	2	[1]	15	[1] / [3]
	Cleistopholis	4	[1]		
	Mezzettia	3	[1]		
	Tetrameranthus	6	[1]		
TMSMFDD	*Dasymaschalon*	21	[1]	171	[1] / [3]
	Desmos	26	[1]		

	Friesodielsia	51	[1]		
	Melodorum	10	[1]		
	Monanthotaxis	56	[1]		
	Sphaerocoryne	3	[1]		
	Toussaintia	4	[1]		
Uvaria	-	187	[1]	187	[19]
Xylopia	-	157	[1]	157	[8]

Ignored diversity

**Ignored genus**	**Nb. of sp. in genus**	**Ref. for sp. content**
Duckeanthus	1	[1]
Boutiquea	1	[1]
Diclinanona	3	[1]
Afroguatteria	2	[1]
Cleistochlamys	1	[1]
Exellia	1	[1]
Gilbertiella	1	[1]
Pyramidanthe	1	[1]
Schefferomitra	1	[1]
Dendrokingstonia	2	[1]
Oncodostigma	2	[1]
Phoenicanthus	2	[1]

Among the 108 putatively monophyletic genera of Annonaceae recognized in the recent phylogenetic classification of Chatrou et al. [1], 19 were not sampled in our dataset, and the monophyly of several of them has not been tested yet. In tribe Ambavioideae, the monophyly of *Ambavia*, *Cleistopholis*, and *Mezzetia* has not been tested before. Consequently, we defined the compartment TCAM to include these three genera as well as *Tetrameranthus* (their closest relative). The monophyly of the resulting compartment has been well supported in the literature [1, 3, 8] and is supported by 100% of PP in our BEAST analyses [see Additional file 2].

Within tribe Bocageeae, genera *Bocagea*, *Cardiopetalum*, and *Froesiodendron* have never been included in a phylogenetic analysis. However, synapomorphies of tribe Bocageeae occur in these three genera, leading Chatrou et al. [1] to argue that they could be securely placed in this monophyletic group. We followed their point of view by incorporating these three genera within one compartment corresponding to the entire tribe Bocageeae. We did not exclude this diversity because it represents 12 % of the species diversity of the defined compartment.

In tribe Duguetieae, *Duckeanthus* has been only included in a morphological cladistic analysis [20]. In their results, the relationships among the remaining genera of the tribe were not in accordance with those supported in recent molecular analyses [1, 3]. Because this genus incorporates only one species, more investigations are needed to confirm its position within the tribe, and a conservative compartment would incorporate more than 99 species (the number of species in Duguetieae), we decided to ignore this species in our analyses.

In tribe Annoneae, the monophyly of *Neostenanthera* and *Asimina* has not been tested before. Consequently, we treated Annoneae as a single compartment in our analyses. This supra-generic compartment has been well supported in the literature [1, 3, 8] and in our BEAST analyses [see Additional file 2]. The genus *Disepalumis* was not sampled in our dataset. The position of this genus as a sister group to *Asimina* has been well supported in the literature [1]. For this reason we included the number of species of this genus in the count of species of our compartment Annoneae. *Boutiquea* was included in tribe Annoneae by Chatrou et al. [1] based on palynological characters, but its phylogenetic position has not been tested in the literature. Because there is only one species in this genus, we preferred to exclude it. The genus *Diclinanona*, also placed in Annoneae, was not sampled in our molecular dating analyses. Its phylogenetic position has been debated in the literature [8, 21]. According to Chatrou et al. [1], who agreed with the results of Richardson et al. [8], a conservative definition for a compartment incorporating this genus would be all Annonoideae except Bocageae. Because this compartment would include 1393 species and *Diclinanona* has only three species, we decided to exclude this genus from our analyses. Because the genus *Deeringothamnus* was considered in the study of Chatrou et al. [1] as a synonym of *Asimina*, we incorporated its species diversity in the count of species of this latter genus.

In tribe Monodoreae, the monophyly of the genus *Mischogyne* has not been tested before. For that reason we defined a compartment, MUMU [see Additional file 2] to include this genus and its sister group including *Uvariodendron*, *Monocyclanthus*, and *Uvariopsis*. This clade has been well supported in the literature [1–3] and received a support value of 100 % of PP in BEAST analyses [see Additional file 2]. In addition, the monophyly of *Asteranthe* has never been tested either. We defined a supra-generic compartment, AHU, to accommodate this genus and its sister

group, the clade of *Hexalobus* plus *Uvariastrum* [see Additional file 2]. This monophyletic group has been well supported in the literature [1–3] and in the present study [see Additional file 2].

In tribe Uvarieae, the monophyly of *Mitrella* and *Toussaintia* has not been tested yet. For this reason, we created two compartments, one including *Mitrella* and its sister group *Fissistigma* (FM), and another including *Toussaintia* and its sister group including *Melodorum*, *Sphaerocoryne*, *Monanthotaxis*, *Friesodielsia*, *Dasymaschalon*, *Desmos* (TMSMFDD, see Additional file 2). Both clades received 100 % of posterior probability in BEAST analyses [see Additional file 2] and have been well supported in the literature [1, 3]. The two species of *Afroguatteria* have never been included in a molecular phylogenetic analysis [1]. Doyle and Le Thomas [22] placed this genus as the sister group to *Uvaria* in a morphological cladistic analysis in which relationships among genera of Annonaceae were not compatible with the current phylogenetic knowledge of the group. However, because a secure placement of the two species of this genus requires more investigation and the genus *Uvaria* contains 187 species, we ignored *Afroguatteria* from our analyses. *Cleistochlamys* and *Gilbertiellia* have never been included in a phylogenetic analysis and their placement within Annonoideae was based on an intuitive approach [1]. Because both of them are monotypic genus and represent less than three percent of any secure compartments in which we could incorporate their diversity, we ignored them from our analyses. Because the monotypic genus *Schefferomitra* has not been placed in a published phylogeny, we preferred to exclude this genus from our analyses. *Exellia,* not included in our molecular dating analyses, has been placed in an unresolved position in Uvariaeae [1]. Because this is a monospecific genus we decided to exclude it. *Pyramidanthe* has been placed with a phylogenetic approach in a clade with *Dasymaschalon*, *Desmos*, *Dielsiothalamnus*, *Fissistigma*, *Friesodielsia*, *Mitrella*, *Monanthotaxis*, *Sphaerocoryne*, *Toussaintia* and *Uvaria*, but the relationships within among these genera remained poorly resolved [23]. Because the incorporation of this monospecific genus would require a conservative compartment consisting of all Uvarieae (at least 415 species), we ignored it.

In tribe Piptostigmateae, *Piptostigma* was shown to be paraphyletic with respect to *Polyceratocarpus* [7], even though Chatrou et al. [1] provisionally maintained the two genera. We defined a compartment including the diversity of the two genera (PP=100% in the present study, see Additional file 2).

In tribe Malmeeae the three genera *Bocageopsis*, *Onychopetalum*, and *Unonopsis* form a clade (PP=100 % in the present study, see Additional file 2), but the relationships among them remain unclear [1, 3, 8, 11]. Because the monophyly of *Onychopetalum* has never been tested before, we defined a compartment including these three genera (BOU, see Additional file 2). In the same tribe, the monophyly of *Pseudomalmea* has never been tested either and its relationships are not well established in the present study. In addition, *Oxandra* has been shown to be polyphyletic even though the support values associated with this polyphyly are low [1, 14]. The least inclusive clade containing all species of *Oxandra* being well supported includes *Mosannona*, *Ruizodendron*, *Ephedranthus*, *Klarobelia*, *Pseudephedranthus*, and *Pseudomalmea* [1, 3, 7, 11; see Additional file 2]. For this reason, we decided to define a compartment including these seven genera (MERKPOP).

Tribe Dendrokingstonieae consists in one genus, *Dendrokingstonia* [1], not sampled in our dataset. Although Chatrou et al. [1] referred to a phylogenetic placement by Chaowasku et al. [24], the methodology used in the latter paper is not clear enough to evaluate the quality of this placement. For this reason and because this genus includes two species only, we decided to exclude it from our analyses.

In tribe Miliuseae, *Fitzalania* was recognized to be valid by Chatrou et al. [1], however a molecular phylogenetic study by Thomas et al. [18] supported a position for this genus nested in *Meiogyne*. Consequently, we included the diversity of *Fitzalania* in *Meiogyne*. In Xue et al. [11], *Polyalthia*, *Enicosanthum*, and *Haplostignanthus* were not monophyletic. The smallest, well supported clade including all the species of these genera also includes all genera of Miliuseae [11]. This tribe has been well supported in the literature [1, 3] and in the present study [see Additional file 2]. We defined a compartment including all the genera of tribe Miliuseae. The monotypic genus *Woodiellantha*, not sampled in our chronograms, was found to be nested in *Orophea* with good support by Chatrou et al. [1] and Richardson et al. [8]. In a more recent study, this taxon was placed in a well-supported clade including several species of *Polyalthia* and *Enicosanthum* [11]. We have counted this species in the count of species of the compartment Miliuseae [see Additional file 2]. The taxonomic status of *Oncodostigma* as an accepted genus or a synonym of *Meiogyne* is not clear and needs further clarification [1]. In their classification, Chatrou et al. [1] did not specify whether the species diversity of this genus was included or not

in the count of species of *Meiogyne*. For this reason and because the genus includes only two species (out of 510 species in our compartment Miliusieae), we have ignored this genus from our analyses. Last, *Phoenicanthus* has never been included in a phylogenetic analysis. For this reason and because there are only two species in this genus, we have also ignored it from our analyses.

Aristolochiaceae + Hydnoraceae

Compartment	Compartment content	Nb. of sp. in genus	Ref. for sp. content	Nb. of sp. in compartment	Ref. for the monophyly of the compartment
Aristolochia	-	400	[25]	400	[26]
Asarum	-	90	[27]	90	[26]
Lactoris	-	1	[28]	1	-
Saruma	-	1	[29]	1	-
Thottea	-	35	[30]	35	[30]

Ignored diversity

Ignored genus	Nb. of sp. in genus	Ref. for sp. content
Hydnora	9	[31]
Prosopanche	2	[32]

All the genera of Aristolochiaceae (incl. *Lactoris*) were sampled in our study. However, Hydnoraceae were excluded (see Molecular dataset section in the materials and methods). Previous studies have placed this parasitic family within Aristolochiaceae [3, 33] and, more recently, Naumann et al. [34] refined its position as sister to subfamily Aristolochioideae. In order to incorporate the nine species of *Hydnora* [31] and the three species of *Prosopanche* [32] in our analyses, we should define a broader compartment to include Hydnoraceae and Aristolochioideae. The species richness of Hydnoraceae represents less than three percent of this compartment. Consequently, we have decided to exclude the family from the count of species.

Atherospermataceae

Compartment	Compartment content	Nb. of sp. in genus	Ref. for sp. content	Nb. of sp. in compartment	Ref. for the monophyly of the compartment

Atherosperma	-	1	[35]	1	-
Daphnandra	-	6	[35]	6	[36]
Doryphora	-	2	[35]	2	[36]
Dryadodaphne	-	3	[35]	3	[36]
Laurelia	-	2	[35]	2	[36]
Laureliopsis	-	1	[35]	1	-
Nemuaron	-	1	[35]	1	-

Calycanthaceae

Compartment	Compartment content	Nb. of sp. in genus	Ref. for sp. content	Nb. of sp. in compartment	Ref. for the monophyly of the compartment
Calycanthus	-	3	[37]	3	[37]
Chimonanthus	-	6	[37]	6	[37]
Idiospermum	-	1	[37]	1	-

Canellaceae

Compartment	Compartment content	Nb. of sp. in genus	Ref. for sp. content	Nb. of sp. in compartment	Ref. for the monophyly of the compartment
Canellaceae	*Canella*	1	[38]	18	[38] / [3]
	Capsicodendron	1	[38]		
	Cinnamodendron	6	[39]		
	Cinnamosma	3	[40]		
	Pleodendron	3	[41]		
	Warburgia	4	[40]		

All genera of the family were sampled in our chronograms. In the phylogenetic analysis of Salazar and Nixon [38], *Cinnamodendron* was paraphyletic and the monophyly of *Cinnasmoma* was not well supported. In addition, deeper relationships in this family were not well supported both in their tree and in the present analyses [see Additional file 2]. Therefore we defined a compartment including all genera of Canellaceae.

Degeneriaceae

Compartment	Compartment content	Nb. of sp. in genus	Ref. for sp. content	Nb. of sp. in compartment	Ref. for the monophyly of the compartment
Degeneria	-	2	[42]	2	Present study

Degeneriaceae include a single genus, *Degeneria*, with two species [42]. The monophyly of this taxon has been tested with *matK* and *ndhF* markers with the separate molecular datasets of Massoni et al. [3]. Because the genus appears to be monophyletic (J. Massoni, unpubl. data), we have defined *Degeneria* as a terminal compartment.

Eupomatiaceae

Compartment	Compartment content	Nb. of sp. in genus	Ref. for sp. content	Nb. of sp. in compartment	Ref. for the monophyly of the compartment
Eupomatia	-	3	[43]	3	[44]

Gomortegaceae

Compartment	Compartment content	Nb. of sp. in genus	Ref. for sp. content	Nb. of sp. in compartment	Ref. for the monophyly of the compartment
Gomortega	-	1	[45]	1	-

Hernandiaceae

Compartment	Compartment content	Nb. of sp. in genus	Ref. for sp. content	Nb. of sp. in compartment	Ref. for the monophyly of the compartment
HIH	*Hazolomania*	1	[46]	45	[46]
	Hernandia	22	[46]		
	Illigera	22	[46]		
Gyrocarpus	-	5	[46]	5	[46]
Sparattanthelium	-	13	[46]	13	[46]

Hernandiaceae incorporate 62 species in five genera [46]. The monophyly of the four non-monospecific genera has been well supported in the literature [46]. *Hernandia* (22 spp.) was not sampled in our chronograms. In Michalak et al. [46], the genera *Hernandia, Hazolomania,* and *Illigera* were found in a clade, but the relationships among them were not resolved. In order to include the diversity of *Hernandia*, we defined a compartment incorporating this genus in addition of *Hazolomania* and *Illigera* (HIH).

Himantandraceae

Compartment	Compartment content	Nb. of sp. in genus	Ref. for sp. content	Nb. of sp. in compartment	Ref. for the monophyly of the compartment
Galbulimima	-	1	[47]	1	-

Lauraceae

Compartment	Compartment content	Nb. of sp. in genus	Ref. for sp. content	Nb. of sp. in compartment	Ref. for the monophyly of the compartment
Lauraceae	*Actinodaphne*	100	[48]	3469	[49] / [50] / [3]
	Adenodaphne	5	[40]		
	Aiouea	19	[51]		
	Alseodaphne	50	[52]		
	Anaueria	1	[53]		
	Aniba	41	[51]		
	Apollonias	2	[52]		
	Aspidostemon	28	[54]		
	Beilschmiedia	250	[55]		
	Caryodaphnopsis	15	[56]		
	Cassytha	20	[57]		
	Chlorocardium	2	[58]		
	Cinnadenia	2	[40]		
	Cinnamomum	250	[59]		
	Clinostemon	2	[53]		
	Cryptocarya	350	[60]		

Dehaasia	35	[52]
Dicypellium	2	[61]
Dodecadenia	2	[40]
Endiandra	129	[40]
Endlicheria	60	[62]
Eusideroxylon	1	[63]
Gamanthera	1	[61]
Hexapora	1	[64]
Hypodaphnis	1	[65]
Iteadaphne	2	[66]
Kubitzkia	2	[61]
Laurus	203	[40]
Licaria	69	[67]
Lindera	121	[40]
Litsea	562	[40]
Machilus	100	[52]
Mezilaurus	21	[68]
Mocinnodaphne	1	[69]
Nectandra	175	[70]
Neocinnamomum	6	[71]
Neolitsea	100	[72]
Nothaphoebe	40	[52]
Ocotea	375	Henk van der Werff (personal communication, 2013: 350-400 species)
Paraia	1	[58]
Parasassafras	1	[40]
Persea	90	[73]
Phoebe	100	[52]
Phyllostemonodaphne	1	[61]
Pleurothyrium	39	[74]
Potameia	21	[75]
Potoxylon	1	[64]
Povedadaphne	1	[76]
Rhodostemonodaphne	41	[77]
Sassafras	3	[78]
Sextonia	2	[79]

Sinapora	1	[80]
Syndiclis	9	[40]
Triadodaphne	3	[64]
Umbellularia	1	[61]
Urbanodendron	3	[61]
Williamodendron	3	[81]
Yasunia	2	[82]

Lauraceae is the largest family within Magnoliidae, uncluding 2500 to 3500 species [57]. The relationships within this clade are still debated in the literature [52, 64, 83]. However, several clades are well supported. This is the case for the Perseeae-Laureeae clade [64], named core Lauraceae by Rohwer and Rudolph [65]. Within the core Lauraceae, Li et al. [52] focused on the phylogeny of the *Persea* group as defined by Rohwer et al. [73]. In addition to the paraphyly of *Alseodaphne* and *Dehaasia*, they found *Persea* to be paraphyletic, with *Apollonias* nested in. In our chronograms, these two genera were sampled and we find them to be nested in a well-supported core Lauraceae clade. However, there is a well-supported conflict among the present study and the literature requiring a larger compartment than core Lauraceae. *Nothaphoebe*, which was nested in this clade in previous studies [52, 73], is found in a different position (outside core Lauraceae) in the present study (see also Massoni et al. [3]). This genus, consisting in about 40 species [52], has never been included in a phylogenetic analysis of Lauraceae as a whole, and a maximum of two species (*Nothaphoebe umbellifora* and *N. semecarpifolia*) have been simultaneously included in the same phylogenetic analysis [52, 73]. In the present study and in Massoni et al. [3], we sampled a different species in order to represent the genus (*N. konishii)*. Because this is the first introduction of this species in a phylogenetic study, and because more than 95 % of the diversity of this genus has never been included in a phylogenetic approach, it is difficult to affirm or disconfirm a misidentification of the taxa used to generate the sequence. Because more investigations are needed to elucidate this question, we preferred to define a conservative compartment including all Lauraceae. Indeed, because an infinitesimal part of the species diversity of Lauraceae has been sampled in previous phylogenetic studies, its distribution in smaller compartments will be problematic.

Magnoliaceae

Compartment	Compartment content	Nb. of sp. in genus	Ref. for sp. content	Nb. of sp. in compartment	Ref. for the monophyly of the compartment
Magnolia	-	223	[84]	223	[84]
Liriodendron	-	2	[85]	2	[84]

Monimiaceae

Compartment	Compartment content	Nb. of sp. in genus	Ref. for sp. content	Nb. of sp. in compartment	Ref. for the monophyly of the compartment
Hortonia	-	1	[86] / [87]	1	-
Mollinedioideae	*Austromatthaea*	1	[88] / [87]	256	[49] / [89] / [3]
	Decarydendron	4	[89]		
	Ephippiandra	7	[89]		
	Faika	1	[87]		
	Grazielanthus	1	[90]		
	Hedycarya	15	[88]		
	Hemmantia	1	[40]		
	Hennecartia	1	[90]		
	Kairoa	3	[87]		
	Kibara	40	[86]		
	Kibaropsis	1	[91]		
	Levieria	7	[89]		
	Macropeplus	4	[92]		
	Macrotorus	1	[91]		
	Matthaea	13	[40]		
	Mollinedia	70	[90]		
	Steganthera	17	[93] / [88]		
	Tambourissa	51	[40]		
	Tetrasynandra	3	[88]		

	Wilkiea	14	[40]		
	Xymalos	1	[87]		
Monimia	-	3	[86]	3	[89]
Palmeria	-	14	[86]	14	[89]
Peumus	-	1	[89]	1	-

Ignored diversity

Ignored genus	Nb. of sp. in genus	Ref. for sp. content
Lauterbachia	1	[89]
Parakibara	1	[89]

Monimiaceae consist of about 280 species in 28 genera. Renner et al. [89] challenged the monophyly of four genera in the family. *Tetrasynandra* was nested within *Steganthera* in their maximum likelihood analysis with moderate support values. The monotypic genus *Grazielanthus* was nested in *Mollinedia*, but the relationships among the species of both genera were not supported. Finally, *Hedycarya* and *Wilkiea* were paraphyletic with good support values involving *kibaropsis*, *Levieria*, and *Kairoa*, *kibara* respectively. In order to take into account the paraphyly of *Wilkiea*, we needed to define a well-supported compartment (PP=100% in the present study) including at least *Wilkiea*, *Kibara*, and *Kairoa* [see Additional file 2], and corresponding to subfamily Mollinedioideae [see Additional file 2]. This clade is well supported in the literature [3, 49, 89]. The monotypic genus *Lauterbachia* discovered in 1899 has not been sampled since this date and the type specimen may have been destroyed [89]. Because it has never been included in a phylogenetic study, and its diversity is negligible, we have decided to ignore this genus. The placement of *Parakibara* (one species) has never been investigated with a phylogenetic approach either. This genus, known only from the type collection, is ignored in the present study. Finally, the last genus not sampled in our chronograms is *Ephippiandra*. In their maximum likelihood phylogeny, Renner et al. [89] placed this taxon with high support values within the core Monimiaceae. Consequently, we incorporated its species diversity (seven species) in count of species of the compartment core Monimiaceae.

Myristicaceae

Compartment	Compartment content	Nb. of sp. in genus	Ref. for sp. content	Nb. of sp. in compartment	Ref. for the monophyly of the compartment
Myristicaceae	*Bicuiba*	1	[85]	476	[85] / [3]
	Brochoneura	3	[94]		
	Cephalosphaera	1	[85]		
	Coelocaryon	4	[85]		
	Compsoneura	12	[85]		
	Doyleanthus	1	[85]		
	Endocomia	4	[85]		
	Gymnacranthera	7	[85]		
	Haematodendron	1	[85]		
	Horsfieldia	104	[85]		
	Iryanthera	25	[85]		
	Knema	95	[85]		
	Mauloutchia	10	[94]		
	Myristica	144	[85]		
	Osteophloeum	2	[85]		
	Otoba	1	[85]		
	Paramyristica	1	[85]		
	Pycnanthus	3	[85]		
	Scyphocephalium	2	[85]		
	Staudtia	1	[85]		
	Virola	54	[85]		

21 genera are currently recognized in Myristicaceae [85]. The phylogenetic relationships among and within genera of this family remain poorly known. In their combined morphological and molecular analysis, Sauquet et al. [85] sampled all 21 genera but sole the monophyly of the two genera *Brochoneura* and *Mauloutchia* was tested. Because the monophyly of all other genera incorporating more than one species has not been tested in the literature and the positions of genera not sampled in our chronograms are unclear in the family, we defined one compartment to include all the species of Myristicaceae [see Additional file 2].

Piperaceae

Compartment	Compartment content	Nb. of sp. in genus	Ref. for sp. content	Nb. of sp. in compartment	Ref. for the monophyly of the compartment
Manekia	-	3	[95]	3	[96]
Peperomia	-	1600	[97] (1500-1750 spp)	1600	[96]
Piper	-	1050	[98]	1050	[96]
Verhuellia	-	3	[99]	3	[100]
Zippelia	-	1	[96]	1	-

Saururaceae

Compartment	Compartment content	Nb. of sp. in genus	Ref. for sp. content	Nb. of sp. in compartment	Ref. for the monophyly of the compartment
Anemopsis	-	1	[101]	1	-
Gymnotheca	-	2	[101]	2	[101]
Houttuynia	-	1	[101]	1	-
Saururus	-	2	[101]	2	[101]

Siparunaceae

Compartment	Compartment content	Nb. of sp. in genus	Ref. for sp. content	Nb. of sp. in compartment	Ref. for the monophyly of the compartment
Siparunaceae	*Glossocalyx*	1	[102]	54	[49] / [103]
	Siparuna	53	[102]		

The genus *Siparuna* was not sampled in our phylogeny. Because the monophyly of the clade including *Glossocalyx* and *Siparuna* has been well supported in the literature [49, 103], we defined a supra-generic compartment including all species of *Glossacalyx* and *Siparuna*.

Winteraceae

Compartment	Compartment content	Nb. of sp. in genus	Ref. for sp. content	Nb. of sp. in compartment	Ref. for the monophyly of the compartment
Drimys	-	6	[104] (between 5 and 7) / [98]	6	[105] / [106]
PZ	*Pseudowintera*	3	[107]	62	[105] / [106]
	Zygogynum	59	[105]		
Takhtajania	-	1	[108]	1	[105] / [106]
Tasmannia	-	36	[105]	36	[105]

The taxonomy within the family varies among authors. The number of genera recognized ranges from eight to five. In the present study, we followed the taxonomic revisions of Vink [109, 110], in which five genera of Winteraceae are recognized. The monophyly of these taxa have been tested and confirmed in Marquínez et al. [105] and Pratt [106]. *Zygogynum* was not sampled in our chronograms. Because in the literature this genus is sister to *Pseudowintera* [105, 106, 111, 112], we incorporated the two genera in a single compartment (PZ, see Additional file 2).

References

1. Chatrou LW, Pirie MD, Erkens RHJ, Couvreur TLP, Neubig KM, Abbott JR, Mols JB, Maas JW, Saunders RMK, Chase MW: **A new subfamilial and tribal classification of the pantropical flowering plant family Annonaceae informed by molecular phylogenetics**. *Bot J Linn Soc* 2012, **169**:5–40.

2. Couvreur TLP, Richardson JE, Sosef MSM, Erkens RHJ, Chatrou LW: **Evolution of syncarpy and other morphological characters in African Annonaceae: A posterior mapping approach**. *Mol Phylogenet Evol* 2008, **47**:302–318.

3. Massoni J, Forest F, Sauquet H: **Increased sampling of both genes and taxa improves resolution of phylogenetic relationships within Magnoliidae, a large and early-diverging clade of angiosperms**. *Mol Phylogenet Evol* 2014, **70**:84–93.

4. Couvreur TLP: **Revision of the African genus *Uvariastrum* (Annonaceae)**. *PhytoKeys* 2014, **33**:1–40.

5. Scharaschkin T, Doyle JA: **Phylogeny and historical biogeography of *Anaxagorea* (Annonaceae) using morphology and non-coding chloroplast sequence data**. *Syst Bot* 2005, **30**:712–735.

6. Pirie MD, Chatrou LW, Erkens RHJ, Maas JW, van der Niet T, Mols JB, Richardson JE: **Phylogeny reconstruction and molecular dating in four Neotropical genera of Annonaceae: the effect of taxon sampling in age estimations**. *Regnum Veg* 2005, **143**:149–174.

7. Couvreur TLP, van der Ham RWJM, Mbele YM, Mbago FM, Johnson DM: **Molecular and morphological characterization of a new monotypic genus of Annonaceae, *Mwasumbia* from Tanzania**. *Syst Bot* 2009, **34**:266–276.

8. Richardson JE, Chatrou LW, Mols JB, Erkens RHJ, Pirie MD: **Historical biogeography of two cosmopolitan families of flowering plants: Annonaceae and Rhamnaceae**. *Philos Trans R Soc Lond B Biol Sci* 2004, **359**:1495–1508.

9. Fero M, Aedo C, Cabezas F, Velayos M: **Taxonomic Revision of *Neostenanthera* (Annonaceae)**. *Syst Bot* 2014, **39**:17–30.

10. Thongpairoj U-S: **Taxonomy and molecular phylogeny of *Artabotrys* R. Brown and palynology of tribe Unoneae (Annonaceae) in Thailand**. Chang Mai University; 2008.

11. Xue B, Su YCF, Thomas DC, Saunders RMK: **Pruning the polyphyletic genus *Polyalthia* (Annonaceae) and resurrecting the genus *Monoon***. *Taxon* 2012, **61**:1021–1039.

12. Surveswaran S, Wang RJ, Su YCF, Saunders RMK: **Generic delimitation and historical biogeography in the early-divergent “ambavioid” lineage of Annonaceae: *Cananga*, *Cyathocalyx* and *Drepananthus***. *Taxon* 2010, **59**:1721–1734.

13. Saunders RMK, Su YCF, Xue B: **Phylogenetic affinities of *Polyalthia* species (Annonaceae) with columellar-sulcate pollen: Enlarging the Madagascan endemic genus *Fenerivia***. *Taxon* 2011, **60**:1407–1416.

14. Pirie MD, Chatrou LW, Mols JB, Erkens RHJ, Oosterhof J: **“Andean-centred” genera in the short-branch clade of Annonaceae: testing biogeographical hypotheses using phylogeny reconstruction and molecular dating**. *J Biogeogr* 2006, **33**:31–46.

15. Erkens RHJ, Chatrou LW, Maas JW, van der Niet T, Savolainen V: **A rapid diversification of rainforest trees (*Guatteria*; Annonaceae) following dispersal from Central into South America.** *Mol Phylogenet Evol* 2007, **44**:399–411.

16. Couvreur TLP: **Monograph of the syncarpous African genera *Isolona* and *Monodora* (Annonaceae).** *Syst Bot Monogr* 2009, **87**:1–150.

17. Pirie MD, Doyle JA: **Dating clades with fossils and molecules: the case of Annonaceae**. *Bot J Linn Soc* 2012, **169**:84–116.

18. Thomas DC, Surveswaran S, Xue B, Sankowsky G, Mols JB, Keßler PJA, Saunders RMK: **Molecular phylogenetics and historical biogeography of the *Meiogyne-Fitzalania* clade (Annonaceae): generic paraphyly and late Miocene-Pliocene diversification in Australasia and the Pacific**. *Taxon* 2012, **61**:559–575.

19. Zhou L, Su YCF, Thomas DC, Saunders RMK: **“Out-of-Africa” dispersal of tropical floras during the Miocene climatic optimum: evidence from *Uvaria* (Annonaceae)**. *J Biogeogr* 2012, **39**:322–335.

20. Chatrou LW, Koek-Noorman J, Maas P: **Studies in Annonaceae XXXVI. The *Duguetia* alliance: where the ways part.** *Ann Missouri Bot Gard* 2000, **87**:234–254.

21. Erkens RHJ, Maas JW, Couvreur TLP: **From Africa via Europe to South America: migrational route of a species-rich genus of neotropical lowland rain forest trees (*Guatteria*, Annonaceae)**. *J Biogeogr* 2009, **36**:2338–2352.

22. Doyle JA, Le Thomas A: **Phylogenetic analysis and character evolution in Annonaceae**. *Bull du Muséum Natl d'histoire Nat Sect B, Adansonia* 1996, **18**:279–334.

23. Zhou L, Su YCF, Chalermglin P, Saunders RMK: **Molecular phylogenetics of *Uvaria* (Annonaceae): relationships with *Balonga*, *Dasoclema* and Australian species of *Melodorum***. *Bot J Linn Soc* 2010, **163**:33–43.

24. Chaowasku T, Kebler PJA, van der Ham RWJM: **A taxonomic revision and pollen morphology of the genus *Dendrokingstonia* (Annonaceae)**. *Bot J Linn Soc* 2012, **168**:76–90.

25. Ohi-Toma T, Sugawara T, Murata H, Wanke S, Neinhuis C, Murata J: **Molecular phylogeny of *Aristolochia* sensu lato (Aristolochiaceae) based on sequences of *rbcL*, *matK*, and phyA genes, with special reference to differentiation of chromosome numbers**. *Syst Bot* 2006, **31**:481–492.

26. Neinhuis C, Wanke S, Hilu KW, Müller K, Borsch T: **Phylogeny of Aristolochiaceae based on parsimony, likelihood, and Bayesian analyses of *trnL-trnF* sequences**. *Plant Syst Evol* 2005, **250**:7–26.

27. Jiang N, Peng X-M, Yu W-B: **Valid Publication of *Asarum longirhizomatosum* (Aristolochiaceae)**. *Novon A J Bot Nomencl* 2011, **21**:190–191.

28. González F, Rudall P: **The questionable affinities of *Lactoris*: evidence from branching pattern, inflorescence morphology, and stipule development**. *Am J Bot* 2001, **88**:2143–2150.

29. González F, Rudall PJ: **Structure and development of the ovule and seed in Aristolochiaceae, with particular reference to *Saruma***. *Plant Syst Evol* 2003, **241**:223–244.

30. Oelschlägel B, Wagner S, Salomo1 K, Sukumaran Pradeep N, Yao TL, Isnard S, Rowe N, Neinhuis C, Wanke S: **Implications from molecular phylogenetic data for systematics, biogeography and growth form evolution of *Thottea* (Aristolochiaceae)**. *Gard Bull Singapore* 2011, **63**:259–275.

31. Bolin JF, Maass E, Musselman LJ: **A New Species of *Hydnora* (Hydnoraceae) from Southern Africa**. *Syst Bot* 2011, **36**:255–260.

32. Ferreira Machado R, Paganucci de Queiroz L: **A new species of *Prosopanche* (Hydnoraceae) from northeastern Brazil**. *Phytotaxa* 2012, **75**:58–64.

33. Nickrent DL, Blarer A, Qiu Y-L, Soltis DE, Soltis PS, Zanis MJ: **Molecular data place Hydnoraceae with Aristolochiaceae**. *Am J Bot* 2002, **89**:1809–1817.

34. Naumann J, Salomo K, Der JP, Wafula EK, Bolin JF, Maass E, Frenzke L, Samain M-S, Neinhuis C, de Pamphilis CW, Wanke S: **Single-copy nuclear genes place haustorial Hydnoraceae within Piperales and reveal a Cretaceous origin of multiple parasitic angiosperm lineages**. *PLoS One* 2013, **8**:e79204.

35. Schodde R: **A monograph of the family Atherospermataceae**. *PhD thesis*. University of Adelaide; 1969.

36. Renner SS, Foreman DB, Murray D: **Timing transantarctic disjunctions in the Atherospermataceae (Laurales): evidence from coding and noncoding chloroplast sequences**. *Syst Biol* 2000, **49**:579–591.

37. Zhou S, Renner SS, Wen J: **Molecular phylogeny and intra- and intercontinental biogeography of Calycanthaceae.** *Mol Phylogenet Evol* 2006, **39**:1–15.

38. Salazar J, Nixon KC: **New discoveries in the Canellaceae in the Antilles: how phylogeny can support taxonomy**. *Bot Rev* 2008, **74**:103–111.

39. De Barros F, Salazar J: ***Cinnamodendron occhionianum*, a New Species of Canellaceae from Brazil**. *Novon A J Bot Nomencl* 2009, **19**:11–14.

40. **The Plant List** [http://www.theplantlist.org/]

41. Hammel BE, Zamora NA: ***Pleodendron costaricense* (Canellaceae), a new species for Costa Rica**. *Lankesteriana* 2005, **5**:211–218.

42. Kubitzki K: **Degeneriaceae**. In *The Families and Genera of Vascular Plants*. Edited by Kubitzki K, Rohwer JG, Bittrich V. Berlin Heidelberg New-York: Springer-Verlag; 1993:290–291.

43. Endress PK: **Early floral development and nature of the calyptra in Eupomatiaceae (Magnoliales)**. *Int J Plant Sci* 2003, **164**:489–503.

44. Kim S, Yoo M-J, Albert VA, Farris JS, Soltis PS, Soltis DE: **Phylogeny and diversification of B-function MADS-box genes in angiosperms: evolutionary and functional implications of a 260-million-year-old duplication.** *Am J Bot* 2004, **91**:2102–2118.

45. Kubitzki K: **Gomortegaceae**. In *The Familiesand Genera of Vascular Plants*. Edited by Kubitzki K, Rohwer JG, Bittrich V. Berlin Heidelberg New-York: Springer-Verlag; 1993:318–320.

46. Michalak I, Zhang L-B, Renner SS: **Trans-Atlantic, trans-Pacific and trans-Indian Ocean dispersal in the small Gondwanan Laurales family Hernandiaceae**. *J Biogeogr* 2010, **37**:1214–1226.

47. Doweld AB, Shevyryova NA: **Carpology, seed anatomy and taxonomic relationships of *Galbulimima* (Himantandraceae)**. *Ann Bot* 1998, **81**:337–347.

48. Zhi-Ming L, Jie L, Xi-Wen L: **Polyphyly of the genus *Actinodaphne* (Lauraceae) inferred from the analyses of nrDNA ITS and ETS sequences**. *Acta Phytotaxon Sin* 2006, **44**:272–285.

49. Renner SS: **Circumscription and phylogeny of the Laurales: evidence from molecular and morphological data**. *Am J Bot* 1999, **86**:1301–1315.

50. Renner SS, Chanderbali AS: **What is the relationship among Hernandiaceae, Lauraceae, and Monimiaceae, and why is this question so difficult to answer?** *Int J Plant Sci* 2000, **161**(Suppl 6):109–119.

51. Kubitzki K, Renner SS: **Lauraceae I (*Aniba* and *Aiouea*)**. *Flora Neotrop* 1982, **31**:1–124.

52. Li L, Li J, Rohwer JG, van der Werff H, Wang Z-H, Li H-W: **Molecular phylogenetic analysis of the *Persea* group (Lauraceae) and its biogeographic implications on the evolution of tropical and subtropical Amphi-Pacific disjunctions**. *Am J Bot* 2011, **98**:1520–1536.

53. Van der Werff H: **A revision of *Mezilaurus* (Lauraceae)**. *Ann Missouri Bot Gard* 1987, **74**:153–182.

54. Van der Werff H: **A revision of the Malagasy endemic genus *Aspidostemon* Rohwer & Richter (Lauraceae)**. *Adansonia* 2006, **28**:7–28.

55. Nishida S: **Revision of *Beilschmiedia* (Lauraceae) in the Neotropics**. *Ann Missouri Bot Gard* 1999, **86**:657–701.

56. Liu B, Yang Y, Ma K: **A new species of *Caryodaphnopsis* airy shaw (Lauraceae) from southeastern Yunnan, China**. *Phytotaxa* 2013, **118**:1–8.

57. Rohwer JG: **Lauraceae**. In *The Families and Genera of Vascular Plants*. Edited by Kubitzki K, Rohwer JG, Bittrich V. Berlin, Heidelberg, New York: Springer-Verlag; 1993:366–391.

58. Rohwer JG, Richter HG, van der Werff H: **Two new genera of Neotropical Lauraceae and critical remarks on the generic delimitation**. *Ann Missouri Bot Gard* 1991, **78**:388–400.

59. Ho K-Y, Hung T-Y: **Cladistic relationships within the genus *Cinnamomum* (Lauraceae) in Taiwan based on analysis of leaf morphology and Inter-Simple Sequence Repeat (ISSR) and Internal Transcribed Spacer (ITS) molecular markers**. *African J Biotechnol* 2011, **10**:4802–4815.

60. Van der Werff H: **A new species and new combinations in *Cryptocarya* from Madagascar**. *Adansonia* 2008, **30**:41–46.

61. Van der Werff H: **A key to the genera of Lauraceae in the new world**. *Ann Missouri Bot Gard* 1991, **78**:377–387.

62. Chanderbali AS: ***Endlicheria* (Lauraceae)**. *Flora Neotrop* 2004, **91**:1–141.

63. Kimoto Y, Utami Na, Tobe H: **Embryology of *Eusideroxylon* (Cryptocaryeae, Lauraceae) and character evolution in the family**. *Bot J Linn Soc* 2006, **150**:187–201.

64. Chanderbali AS, van der Werff H, Renner SS: **Phylogeny and historical biogeography of Lauraceae: evidence from the chloroplast and nuclear genomes**. *Ann Missouri Bot Gard* 2001, **88**:104–134.

65. Rohwer JG, Rudolph B: **Jumping genera: the phylogenetic positions of *Cassytha*, *Hypodaphnis*, and *Neocinnamomum* (Lauraceae) based on different analyses of *trnK* intron sequences**. *Ann Missouri Bot Gard* 2005, **92**:153–178.

66. Li J, Christophel DC: **Systematic relationships within the *Litsea* complex (Lauraceae): a cladistic analysis on the basis of morphological and leaf cuticle data**. *Aust Syst Bot* 2000, **13**:1–13.

67. Van der Werff H: **Nine new species of *Licaria* (Lauraceae) from Tropical America**. *Harvard Pap Bot* 2009, **14**:145–159.

68. Alves FM, van der Werff H, Souza VC: ***Mezilaurus introrsa* (Lauraceae), a new species from Colombia**. *Phytotaxa* 2012, **48**:29–32.

69. Hernandez FGL: ***Mocinnodaphne*, un genero nuevo de la familia Lauraceae en la flora de Mexico**. *Acta Botánica Mex* 1995, **32**:25–32.

70. Wagner de Oliveira C, Henriques Callado C, Marquete O: **Anatomia do lenho de espécies do gênero *Nectandra* Rol. ex Rottb. (Lauraceae)**. *Rodriguésia* 2001, **52**:125–134.

71. Wang Z, Li J, Conran JG, Li H: **Phylogeny of the Southeast Asian endemic genus *Neocinnamomum* H. Liu (Lauraceae)**. *Plant Syst Evol* 2010, **290**:173–184.

72. Li L, Li J, Conran JG, Li X-W, Li) (H.-W.: **Phylogeny of *Neolitsea* (Lauraceae) inferred from Bayesian analysis of nrDNA ITS and ETS sequences**. *Plant Syst Evol* 2007, **269**:203–221.

73. Rohwer JG, Li J, Rudolph B, Schmidt SA, van der Werff H, Li H: **Is *Persea* (lauraceae) monophyletic? evidence from nuclear ribosomal ITS sequences**. *Taxon* 2009, **58**:1153–1167.

74. Van der Werff H: **A revision of the genus *Pleurothyrium* (Lauraceae)**. *Ann Missouri Bot Gard* 1993, **80**:39–118.

75. Van der Werff H: **Studies in Malagasy Lauraceae II: new taxa**. *Novon* 1996, **6**:463–475.

76. Burger WC: **A new genus of Lauraceae from Costa Rica, with comments on problems of generic and specific delimitation within the family**. *Brittonia* 1988, **40**:275.

77. Madriñán S: ***Rhodostemonodaphne* (Lauraceae)**. *Flora Neotrop* 2004, **92**:1–102.

78. Nie Z-L, Wen J, Sun H: **Phylogeny and biogeography of *Sassafras* (Lauraceae) disjunct between eastern Asia and eastern North America**. *Plant Syst Evol* 2007, **267**:191–203.

79. Van der Werff H: ***Sextonia*, a New Genus of Lauraceae from South America**. *Novon* 1997, **7**:436–439.

80. Li J, Xia N, Li X: ***Sinopora*, A new genus of Lauraceae from South China**. *Novon* 2008, **18**:199–201.

81. Van der Werff H: **A new species of *Williamodendron* (Lauraceae) from Southern Brazil**. *Novon* 1991, **1**:6–8.

82. Van der Werff H, Nishida S: ***Yasunia* (Lauraceae), a new genus with two species from Ecuador and Peru**. *Novon A J Bot Nomencl* 2010, **20**:493–502.

83. Rohwer JG: **Toward a phylogenetic classification of the Lauraceae: evidence from *matK* sequences**. *Syst Bot* 2000, **25**:60–71.

84. Kim S, Suh Y: **Phylogeny of Magnoliaceae based on ten chloroplast DNA regions**. *J Plant Biol* 2013, **56**:290–305.

85. Sauquet H, Doyle JA, Scharaschkin T, Borsch T, Hilu KW, Chatrou LW, Le Thomas A: **Phylogenetic analysis of Magnoliales and Myristicaceae based on multiple data sets: implications for character evolution**. *Bot J Linn Soc* 2003, **142**:125–186.

86. Renner SS: **Variation in diversity among Laurales, Early Cretaceous to present**. In *Plant Diversity and Complexity Patterns: Local, Regional and Global Dimensions.* Edited by Friis I, Balslev H. Royal Danish Academy of Sciences and Letters; 2005:441–458.

87. Renner SS, Takeuchi WN: **A phylogeny and revised circumscription for *Kairoa* (Monimiaceae), with the description of a new species from Papua New Guinea**. *Harvard Pap Bot* 2009, **14**:71–81.

88. Sampson FB: **Pollen morphology and ultrastructure of Australian Monimiaceae - *Austromatthaea*, *Hedycarya*, *Kibara*, *Leviera*, *Steganthera* and *Tetrasynandra***. *Grana* 1997, **36**:135–145.

89. Renner SS, Strijk JS, Strasberg D, Thébaud C: **Biogeography of the Monimiaceae (Laurales): a role for East Gondwana and long-distance dispersal, but not West Gondwana**. *J Biogeogr* 2010, **37**:1227–1238.

90. Peixoto AL, Pereira-Moura MVL: **A new genus of Monimiaceae from the Atlantic Coastal Forest in South-Eastern Brazil**. *Kew Bull* 2008, **63**:137–141.

91. Renner SS: **Phylogenetic affinities of Monimiaceae based on cpDNA gene and spacer sequences**. *Perspect Plant Ecol Evol Syst* 1998, **1**:61–77.

92. Costa FF, Lima HRP, Cunha M Da, Santos I da S: **Leaf anatomy and histochemistry of *Macropeplus dentatus* (Perkins) I. Santos & Peixoto and *Macropeplus ligustrinus* (Tul.) Perkins (Monimiaceae)**. *Acta Bot Brasilica* 2010, **24**:852–861.

93. Takeuchi WN: **An unusual new species of *Steganthera* (Monimiaceae) from Papua New Guinea**. *Kew Bull* 2001, **56**:995–998.

94. Doyle JA, Sauquet H, Scharaschkin T, Le Thomas A: **Phylogeny, molecular and fossil dating, and biogeographic history of Annonaceae and Myristicaceae (Magnoliales)**. *Int J Plant Sci* 2004, **165**(suppl 4):55–67.

95. Schubert HK, Taylor MS, Smith JF, Bornstein AJ: **A Systematic Revision of the Genus *Manekia* (Piperaceae)**. *Syst Bot* 2012, **37**:587–598.

96. Wanke S, Jaramillo MA, Borsch T, Samain M-S, Quandt D, Neinhuis C: **Evolution of Piperales-*matK* gene and *trnK* intron sequence data reveal lineage specific resolution contrast.** *Mol Phylogenet Evol* 2007, **42**:477–497.

97. Wanke S, Samain M-S, Vanderschaeve L, Mathieu G, Goetghebeur P, Neinhuis C: **Phylogeny of the genus *Peperomia* (Piperaceae) inferred from the *trnK*/*matK* region (cpDNA).** *Plant Biol* 2006, **8**:93–102.

98. Mabberley DJ: *Mabberley's Plant-Book*. 3rd edition. Cambridge, UK: Cambridge University Press; 1021.

99. Samain M-S, Mathieu G, Wanke S, Neinhuis C, Goetghebeur P: ***Verhuellia* revisited-unravelling its intricate taxonomic history and a new subfamilial classification of Piperaceae**. *Taxon* 2008, **57**:583–587.

100. Wanke S, Vanderschaeve L, Mathieu G, Neinhuis C, Goetghebeur P, Samain M-S: **From forgotten taxon to a missing link? The position of the genus *Verhuellia* (Piperaceae) revealed by molecules.** *Ann Bot* 2007, **99**:1231–1238.

101. Meng S-W, Douglas AW, Li D-Z, Chen Z-D, Liang H-X, Yang J-B: **Phylogeny of Saururaceae based on morphology and five regions from three plant genomes**. *Ann Missouri Bot Gard* 2003, **90**:592–602.

102. Renner SS, Hausner G: **Siparunaceae**. New York: New York Botanical Garden Press Department; 247.

103. Renner SS, Won H: **Repeated Evolution of Dioecy from Monoecy in Siparunaceae (Laurales)**. *Syst Biol* 2001, **50**:700–712.

104. Ruiz E, Toro O, Crawford DJ, Stuessy TF, Negritto MA, Baeza C, Becerra J: **Phylogenetic relationships among Chilean species of *Drimys* (Winteraceae) based on ITS sequences and insertion/deletion events**. *Gayana Botánica* 2008, **65**:220–228.

105. Marquínez X, Lohmann LG, Salatino MLF, Salatino A, González F: **Generic relationships and dating of lineages in Winteraceae based on nuclear (ITS) and plastid (*rpS16* and *psbA-trnH*) sequence data.** *Mol Phylogenet Evol* 2009, **53**:435–449.

106. Pratt SJ: **Evolution of the genera *Vitex* (Lamiaceae) and *Zygogynum* (Winteraceae) on New Caledonia**. *Master thesis*. The University of Waikato; 2013

107. Lloyd DG, Wells MS: **Reproductive biology of a primitive angiosperm, *Pseudowintera colorata* (Winteraceae), and the evolution of pollination systems in the Anthophyta**. *Plant Syst Evol* 1992, **181**:77–95.

108. Suh Y, Thien LB, Reeve HE, Zimmer EA: **Molecular evolution and phylogenetic implications of internal transcribed spacer sequences of ribosomal DNA in Winteraceae**. *Am J Bot* 1993, **80**:1042–1055.

109. Vink W: **The Winteraceae of the Old World V. *Exospermum* links *Bubbia* to *Zygogynum*.** *Blumea* 1985, **31**:39–55.

110. Vink W: **Taxonomy in Winteraceae.** *Taxon* 1988, **37**:691–698.

111. Karol KG, Suh Y, Schatz GE, Zimmer EA: **Molecular evidence for the phylogenetic position of *Takhtajania* in the Winteraceae: inference from nuclear ribosomal and chloroplast gene spacer sequences**. *Ann Missouri Bot Gard* 2000, **87**:414–432.

112. Doust AN, Drinnan AN: **Floral development and molecular phylogeny support the generic status of *Tasmannia* (Winteraceae).** *Am J Bot* 2004, **91**:321–331.
